# Regional Control of Chromosome Segregation in *Pseudomonas aeruginosa*

**DOI:** 10.1371/journal.pgen.1006428

**Published:** 2016-11-07

**Authors:** Valentine Lagage, Frédéric Boccard, Isabelle Vallet-Gely

**Affiliations:** Institute for Integrative Biology of the Cell (I2BC), CEA, CNRS, Univ. Paris‐Sud, Université Paris‐Saclay, Gif‐sur‐Yvette cedex, France; A*STAR, SINGAPORE

## Abstract

Chromosome segregation in bacteria occurs concomitantly with DNA replication, and the duplicated regions containing the replication origin *oriC* are generally the first to separate and migrate to their final specific location inside the cell. In numerous bacterial species, a three-component partition machinery called the ParABS system is crucial for chromosome segregation. This is the case in the gammaproteobacterium *Pseudomonas aeruginosa*, where impairing the ParABS system is very detrimental for growth, as it increases the generation time and leads to the formation of anucleate cells and to *oriC* mispositioning inside the cell. In this study, we investigate *in vivo* the ParABS system in *P*. *aeruginosa*. Using chromatin immuno-precipitation coupled with high throughput sequencing, we show that ParB binds to four *parS* site located within 15 kb of *oriC in vivo*, and that this binding promotes the formation of a high order nucleoprotein complex. We show that one *parS* site is enough to prevent anucleate cell formation, therefore for correct chromosome segregation. By displacing the *parS* site from its native position on the chromosome, we demonstrate that *parS* is the first chromosomal locus to be separated upon DNA replication, which indicates that it is the site of force exertion of the segregation process. We identify a region of approximatively 650 kb surrounding *oriC* in which the *parS* site must be positioned for chromosome segregation to proceed correctly, and we called it “competence zone” of the *parS* site. Mutant strains that have undergone specific genetic rearrangements allow us to propose that the distance between *oriC* and *parS* defines this “competence zone”. Implications for the control of chromosome segregation in *P*. *aeruginosa* are discussed.

## Author Summary

Accurate transmission of the genetic information relies on replication and segregation, two processes essential to all living organisms. In bacteria, these processes occur concomitantly. Replication of the bacterial circular chromosome initiates at a single specific sequence called *oriC*, and proceed bi-directionally along the chromosome arms. A partition system called ParABS is involved in chromosome segregation in many bacteria. It involves the binding of the ParB protein to *parS* sequences, which are often found in the close vicinity of *oriC*. The importance of this system for chromosome segregation varies according to species, ranging from essential to dispensable. In *Pseudomonas aeruginosa*, an important opportunistic pathogen, the ParABS system plays an important role in chromosome segregation, as mutants affected in this system present a severe growth defect as well as anucleate cells formation, but is not essential. In this study, we characterize the activity of the different determinants of the ParABS system in *P*. *aeruginosa* and demonstrate that it is critical for the *parS* site to be located close to *oriC*, which suggest that the timing of separation of regions close to *oriC* after replication is important, and that it could be a function of the ParABS system to keep this timing.

## Introduction

Most bacteria possess a single chromosome, circular and replicated bi-directionally from a specific sequence called *oriC*. It must be highly compacted to fit inside the bacterial cell, which is typically one thousand times smaller than it is when extended. Despite recent advances, the mechanisms by which the two copies of the chromosome are segregated in daughter cells concomitantly with replication—allowing faithful transmission of genetic material—remains mostly a mystery. Diverse forces might be involved, including DNA replication [1], intranucleoid pushing forces resulting from radial confinement [2], or forces originating from polymer properties (that vary according to different models that consider the chromosome as either a self-avoiding [3] or a self-adherent polymer [4]).

In bacteria, two main molecular actors that are involved in chromosome segregation have been characterized: the structural maintenance of chromosome (SMC) complex and the ParABS system. SMC proteins are ubiquitous from eukaryotes to prokaryotes, and are only absent in a few bacterial species [5]. In contrast, the ParABS system is specific to bacteria, and was found in 70% of the sequenced species in 2007 [6]. It comprises three components. The ParB protein binds specifically to *parS* sites (sometimes compared to centromeric sequences) to form a nucleoprotein complex. ParA is a Walker A-type ATPase thought as the “motor” that provides the force for the segregation of the nucleoprotein complex [7]. Briefly, interaction of ParA-ATP dimer bound aspecifically to DNA with the ParB-*parS* nucleoprotein complex induces its ATPase activity and its release from DNA, which is thought to pull the ParB/*parS* complex via a diffusion-Ratchet mechanism [8]. This model was further elaborated by the recent proposition that the elasticity of chromosomal DNA could contribute to the directional transport of the ParB-*parS* nucleoprotein complex across a ParA-ATP gradient [9]. Moreover, two different models have been proposed regarding the molecular basis involved in the ParB-*parS* nucleoprotein complex formation, either by ParB from *Bacillus subtilis* (the “spreading and bridging” model, [10,11]) or by ParB from the F plasmid (the “nucleation and caging” model, [12]). Both models require the ability of ParB to also bind DNA non-specifically [13].

Although the *parS* sites exhibit an unusually high degree of sequence conservation and close proximity to *oriC* in the vast majority of bacteria, the copy number varies among species from 1 to more than 20 [6]. A functional link between the ParABS system and the SMC complex has been demonstrated in *B*. *subtilis* and *Streptococcus pneumoniae*, where the ParB-*parS* complex recruits SMC to the *oriC* region, thereby allowing correct chromosome segregation [14–16]. This recruitment is thought to depend on ParB ability to bridge DNA, a phenomenon previously described as “spreading” [10], but may also require a specific interaction of SMC with ParB [17,18].

The gamma-proteobacterium *Pseudomonas aeruginosa* is an ubiquitous opportunistic pathogen responsible for nosocomial infections and for the morbidity of Cystic Fibrosis patients. The large size of its genome (6.3 Mb) results from genetic complexity rather than gene duplication, and allows this bacterium to colonize diverse niches [19,20]. It was previously shown that a ParABS system and an SMC complex participate in chromosome segregation in *P*. *aeruginosa* [21–26]. Ten *parS* sites scattered along the chromosome have been proposed, based on sequence homology and *in vitro* binding experiments [21,27]; however, another bioinformatics study predicted only 4 *parS* close to *oriC* in *P*. *aeruginosa* [6]. We previously showed that *P*. *aeruginosa* PAO1 chromosome is globally oriented from the old pole of the cell to the division plane/new pole along the *oriC*-*dif* axis, with the *oriC* region positioned around the 0.2/0.8 relative cell length in a ParA and ParB dependent manner [26].

To better understand the molecular function of the ParABS system in *P*. *aeruginosa*, we used here an *in vivo* approach to identify and characterize the activity of its different determinants. We show that ParB binds *in vivo* to 4 *parS* sites located close to *oriC*, and that one of these *parS* is sufficient for proper chromosome segregation. Using cells that carry only one *parS* site, and displacing this *parS* site from its native position, we show that *parS* is bound by ParB independently of its location on the chromosome, and positioned near the 0.2/0.8 relative cell length in a ParA dependent manner. It is the first chromosomal locus to be separated after replication, indicating that it is the site of force exertion of the segregation process. Moreover, we identify a region of approximately 650 kb surrounding *oriC* in which the *parS* site must be located for chromosome segregation to proceed correctly (as assessed by quantification of anucleate cell in growing cultures), suggesting a regional control of chromosome segregation in *P*. *aeruginosa*. We further provide evidence that efficient chromosome segregation requires proximity between *parS* and *oriC*, suggesting coordination between ParABS mediated chromosome segregation and the replication process.

## Results

### Identification of ParB binding sites *in vivo*

**Fig 1:**
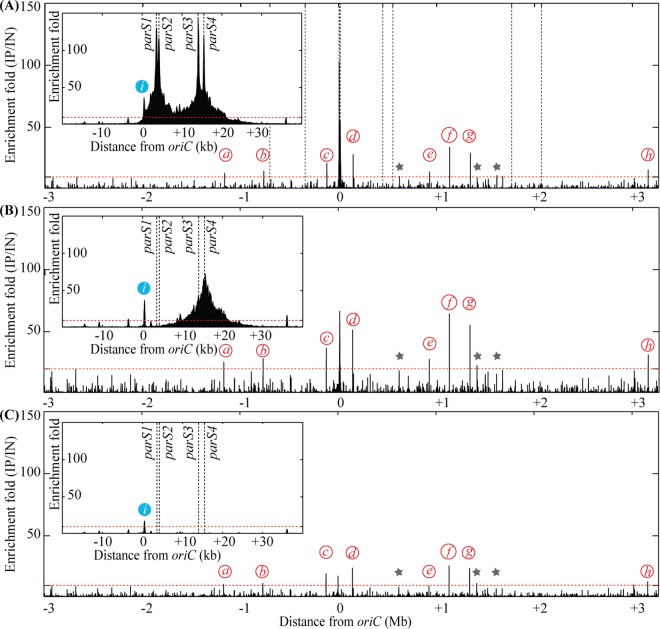
**Chromatin Immunoprecipitation of ParB-3xFLAG,** in the wild type background (strain IVGB379) (A), in the Δ*parS123* background (strain VLB3) (B) and in the Δ*parS1234* background (strain VLB4) (C). Enrichment folds between the immunoprecipitated (IP) and the input (IN) fractions are represented for each base of the genome, according to its distance from *oriC*. The insets represent a zoom of the region containing the four *parS* sites bound by ParB. Dashed vertical lines represent the position of the proposed *parS* sites from [21]. Red italicized letters indicated ParB accessory binding sites, and blue italicized letters represents the promoter region of *dnaA*. Grey stars indicate peaks that are less prominent but still found in the different genetic backgrounds. The red dotted lines indicate the significant enrichment. Precise description of the different peaks is given in S1 Table.

It was previously reported that the *P*. *aeruginosa* ParABS system plays a role in chromosome segregation [26,27]. However, there has been controversy about number of *parS* sites on the genome. To identify ParB binding sites *in vivo*, we replaced the chromosomal copy of the *parB* gene by a gene encoding a 3xFLAG tagged version of ParB (S1A Fig). The resulting strain behaves like the wild type strain (same generation time of 47 minutes and same amount of anucleate cells, less than 1%, S1B Fig). We then analyzed the positioning of two chromosomal loci inside growing cells using fluorescent microscopy. In minimal medium supplemented with citrate, chromosomal tags located near *oriC* and *dif* present the same localization pattern in the PAO1 ParB-3xFLAG strain and in the wild type PAO1 strain (S1C Fig), indicating that the ParB-3xFLAG is fully functional. We performed Chromatin Immunoprecipitation of ParB-3xFLAG followed by high throughput sequencing (ChIP-seq, see methods). Results presented in Fig 1A and S1 Table show that *in vivo*, ParB is mostly bound to four of the ten *parS* sites previously proposed (*parS1* and *parS4* (TGTTCCACGTGGAACC), and *parS2* and *parS3* (TGTTCCACGTGGAACA) from [21], which were also predicted as *parS* sites by [6]). Strong enrichment was found in more than 20 kb surrounding these 4 *parS* sites, which is consistent with the “spreading” phenomenon described for ParB binding to *parS* sites in other bacteria [10,12,13]. This suggests that these four *parS* site are included in a large nucleoprotein complex formed by ParB. In contrast with previous *in vitro* results, ParB does not bind significantly *in vivo* to the other proposed *parS* sequences, which present two mismatches compared to the *parS1*/*parS2* sequences. We thus propose the TGTTCCACGTGGAACM sequence as *P*. *aeruginosa parS* site. This sequence is found in 4 occurrences in *P*. *aeruginosa* genome, and no additional occurrence is found when only one mismatch is allowed. It is interesting to note that ChIP-seq analysis revealed eight additional secondary ParB binding sites that do not contain putative *parS* sequences (Fig 1 and S1 Table). However, the enrichment profile at these sites is different from that obtained with the four *parS* sites involved in chromosome segregation, as no “spreading” was observed (S2A Fig). As a control, we performed ChIP-seq experiments in a strain lacking the ParB-3xFLAG protein, and no enrichment was observed around the *parS* sites and the ParB secondary binding sites mentioned above (S2B, S2C and S2D Fig).

### A single *parS* site is sufficient to sustain chromosome segregation

To study the role of ParB binding sites in chromosome segregation and positioning inside the cell, we inactivated the *parS* sites bound by ParB. Several point mutations were introduced in *parS1* and *parS2* and unmarked deletion of *parS3* and *parS4* were performed (see S1 Text).

**Fig 2:**
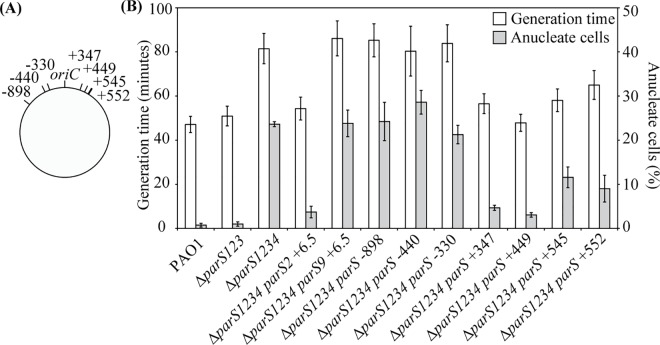
Impact of *parS* site location on the chromosome on generation time and amount of anucleate cell found in liquid cultures. (A) Schematic representation of the position of the *parS* sites introduced in the Δ*parS1234* mutant. (B) Generation times (white bars, scale on the left axis) and percentage of anucleate cell (grey bars, scale on the right axis) of the different strains used in this study. Histograms and error bars represent the mean and standard deviation for at least three independent experiments. Strains IVGB469 (Δ*parS123*), VLB1 (Δ*parS1234*), VLB63 (Δ*parS1234 parS2* +6.5), VLB62 (Δ*parS1234 parS9* +6.5), VLB69 (Δ*parS1234 parS* -898), VLB70 (Δ*parS1234 parS* -440), IVGB481 (Δ*parS1234 parS* -330), VLB66 (Δ*parS1234 parS* +347), IVGB479 (Δ*parS1234 parS* +449), VLB73 (Δ*parS1234 parS* +545) and IVGB480 (Δ*parS1234 parS* +552)were used.

No obvious growth defect could be observed when *parS1*, *parS2* and *parS3* were inactivated (alone or in combination), in contrast to when the four *parS* sites were inactivated, which is consistent with results from Jecz and colleagues [27]. Therefore, we focused our study on the mutant presenting only one functional *parS* site (Δ*parS123*) and the mutant defective in all four *parS* sites (Δ*parS1234)*. We first measured their growth ability in minimal medium supplemented with glucose and casamino acids. The generation time of the Δ*parS123* mutant was similar to that of the wild type strain (50 and 47 minutes, respectively, Fig 2 and S3 Fig), whereas the generation time of the Δ*parS1234* mutant was considerably increased (81 minutes), as observed for the Δ*parB* mutant [26]. The amount of anucleate cells was also low for the wild type strain and the Δ*parS123* mutant (0.7 and 1% respectively, Fig 2), in contrast to the Δ*parS1234* mutant (approximately 25%). In agreement with these results, the positioning of chromosomal tags located near *oriC* and *dif* was the same in the wild type strain and in the Δ*parS123* mutant (S4 Fig). In contrast, it was strongly affected in the Δ*parS1234* mutant (S4 Fig), like in the Δ*parB* and Δ*parA* mutants (previously described in [26]).

To further confirm that a single *parS* site bound by ParB is sufficient to sustain proper chromosome segregation in *P*. *aeruginosa*, we re-introduced a *parS2* site 6.5 kb away from *oriC* (downstream of *gyrB)* in the Δ*parS1234* mutant. The resulting strain (Δ*parS1234 parS2* +6.5) presents a generation time and an amount of anucleate cells only slightly higher than the wild type strain (Fig 2). We also introduced a sequence that is not efficiently bound by ParB *in vivo* (TCTTCCTCGTGGAACA, referred as *parS9* in [21] and [27]) at the exact same location. The resulting strain (Δ*parS1234 parS9* +6.5) presents a generation time and an amount of anucleate cells similar to the Δ*parS1234* mutant (Fig 2). These results indicate that a single *parS* site bound by ParB is enough to sustain proper chromosome segregation, and that a *parS*-derived sequence containing 2 mismatches from the consensus sequence TGTTCCACGTGGAACM is not, probably due to the fact that it is not recognized by ParB *in vivo*.

### ParB secondary binding sites are not sufficient for correct chromosome segregation

To characterize ParB binding to *P*. *aeruginosa* chromosome in the Δ*parS123* and Δ*parS1234* mutants, we replaced the chromosomal copy of the *parB* gene by a gene encoding the 3xFLAG tagged version of ParB and performed ChIP-seq experiments. Results presented in Fig 1B and 1C and S1 Table indicate that more than 8 kb surrounding the *parS4* site are enriched in the Δ*parS123* mutant, whereas no enrichment could be detected around the localization of the *parS* sites in the Δ*parS1234* mutant. This suggests that a nucleoprotein complex is formed by ParB from a single *parS*. Moreover, the secondary sites identified in the wild type strain were also bound by ParB in the Δ*parS123* and Δ*parS1234* mutants. An additional secondary site upstream of *dnaA* was identified. This site is also present in the wild type strain, but it is included in the large region enriched around the *parS* sites (Fig 1A). Considering that the chromosome segregation defect of the Δ*parS1234* mutant is similar to that of the Δ*parB* mutant, the fact that ParB is bound to the secondary sites in the Δ*parS1234* mutant suggests that they are not sufficient for correct chromosome segregation. Moreover, no binding of ParB to the *parS* putative sequences identified *in vitro* in [21] and [27] was observed in the Δ*parS123* and Δ*parS1234* mutants, indicating that even in the absence of the major ParB binding sites, these sequences are not bound by ParB *in vivo*.

### The position of *parS* on the chromosome is critical for efficient chromosome segregation

To investigate the impact of the *parS* site position on chromosome segregation, we reintroduced a *parS* site at different locations in a Δ*parS1234* mutant. We could not obtain and/or propagate strains with a *parS* site located 1 or 1.5 Mb from *oriC* on the right replichore (in contrast to what was described with the PAO1161 strain [27]). However, we could generate strains with a *parS* site located between 898 kb from *oriC* on the left replichore to 550 kb from *oriC* on the right one (Fig 2A). We analyzed growth rate and chromosome segregation in these strains, by measuring the generation time and the amount of anucleate cells present in liquid culture in minimal medium supplemented with glucose and casamino acids. Results are shown in Fig 2B. Compared to the Δ*parS1234* mutant, strains Δ*parS1234 parS* -898, Δ*parS1234 parS* -440 and Δ*parS1234 parS* -330 (which carry a *parS* site 898, 440 and 330 kb from *oriC* on the left replichore, respectively, Fig 2A) present a similar generation time (more than 80 minutes), and a similar amount of anucleate cells (more than 20%). In contrast, generation times of strains Δ*parS1234 parS* +347, Δ*parS1234 parS* +449, Δ*parS1234 parS* +545 and Δ*parS1234 parS* +552 (which carries a *parS* site on the right replichore) were significantly different from the generation time of the Δ*parS1234* mutant, closer to the generation time of the Δ*parS123* mutant (56, 48, 58 and 65 minutes respectively). In addition, the amount of anucleate cells are significantly reduced in these strains compared to the Δ*parS1234* mutant (4, 2, 11 and 9% respectively, compared to 25% for the Δ*parS1234* mutant), even if these amounts are significantly higher than what is observed in the Δ*parS123* mutant (less than 1%). These results indicate that the efficiency of chromosome segregation depends on the location of the *parS* site on the chromosome, suggesting a regional control of this process.

### ParB binding to *parS* is independent of ParA and of the *parS* location on the chromosome

To investigate the apparent difference in functionality of the *parS* site depending of its location on the chromosome, we first tested its ability to be bound by ParB. We built a functional GFP-ParB fusion (see S1 Text) that we used to visualize ParB in different genetic backgrounds. The gene encoding this fusion is expressed from a plasmid (called pPSV38-NGFP-ParB). It is under the control of an IPTG-inducible *lac*UV5 promoter flanked by two *lac* operators, strictly repressed in *P*. *aeruginosa* in absence of IPTG ([28] and S. L. Dove personal communication). This pPSV38-NGFP-ParB plasmid was introduced in different strains containing a native copy of *parB*. We used the minimal amount of IPTG to visualize this fusion without interfering with the cell physiology (overexpression of *parB* is toxic in *P*. *aeruginosa*, [21]). The ability of GFP-ParB to form foci in these strains was assessed using fluorescent microscopy to visualize the GFP fusion. In the Δ*parS123* mutant, more than 90% of the cells contained 2 foci (S2 Table), whereas no visible focus could be observed in the Δ*parS1234* mutant. This indicates that foci formation depends on ParB binding to a *parS* site, and probably of the formation of a nucleoprotein complex (illustrated by spreading of ParB around the *parS* site, see above). Indeed, secondary sites are bound by ParB in the Δ*parS1234* mutant (see above), but this binding in the absence of spreading does not result in visible foci. Also, no focus could be observed when the pPSV38-NGFP-ParB plasmid was introduced in the Δ*parS1234 parS9* +6.5 mutant (containing only a *parS*-derived sequence with 2 mismatches from the consensus sequence).

Strikingly, when the pPSV38-NGFP-ParB plasmid was introduced in strains containing a *bona fide parS* site at different locations on the chromosome (Δ*parS1234 parS* +347, Δ*parS1234 parS* +552 and Δ*parS1234 parS* -898) or in the Δ*parA* mutant, a majority of cells contained foci when observed using fluorescent microscopy (S2 Table). We noticed that the proportion of cells without visible fluorescent focus was higher in the Δ*parS1234 parS* -898 and in the Δ*parA* mutant than in the wild type strain, which is in agreement with the higher proportion of anucleate cells observed with these strains. These results indicate that, in contrast with efficient chromosome segregation, ParB binding to *parS* is not dependent on ParA and on the *parS* position on the chromosome.

### Localization of the ParB/*parS* complex near the 0.2/0.8 relative cell length is dependent on ParA but not on *parS* location on the chromosome

**Fig 3:**
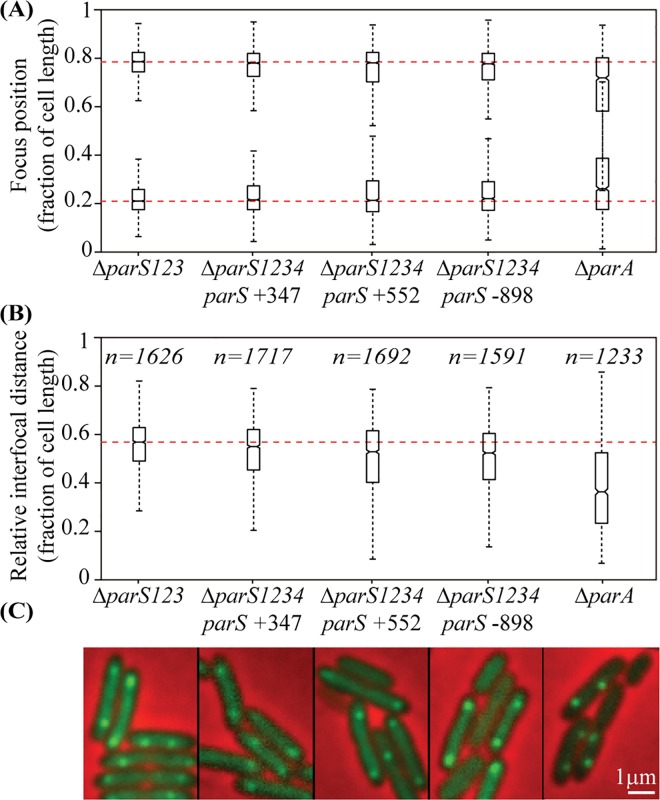
ParB binding to *parS* in the presence of ParA induces its positioning to the 0.2/0.8 relative cell length. The pPSV38-NGFP-ParB plasmid was introduced in different strains, and NGFP-ParB localization was observed in cells grown in minimal medium supplemented with citrate. Foci numbers for each strain are indicated in S2 Table. (A) represents the localization of the 2 foci in cells (randomly oriented) containing two foci, for each genetic background, whereas (B) represents the distance between the two foci. Boxplot representations are used, indicating the median (horizontal bar), the 25th and the 75^th^ percentile (open box) and the rest of the population except for the outliers (whiskers). Outliers are defined as 1.5×*IQR* or more above the 75^th^ percentile or 1.5×*IQR* or more below the first 25th percentile quartile. Representative images are shown in (C). Scale bar is indicated. Raw data are presented in S5 Fig. Strains IVGB469 (Δ*parS123*), VLB66 (Δ*parS1234 parS* +347), IVGB480 (Δ*parS1234 parS* +552), VLB69 (Δ*parS1234 parS* -898) and IVGB317 (Δ*parA*) were used.

The 2 fluorescent foci observed in the Δ*parS123* mutant containing the pPSV38-NGFP-ParB plasmid are positioned close to the 0.2/0.8 relative cell length, with an average interfocal distance of 0.6 relative cell length (Fig 3). Strikingly, when the pPSV38-NGFP-ParB plasmid was introduced in the Δ*parS1234 parS* +347, Δ*parS1234 parS* +552 and Δ*parS1234 parS* -898 strains (presenting 4%, 9% and 25% of anucleate cells, respectively), ParB positioning was not affected, remaining close to the 0.2/0.8 relative cell length independently of the *parS* site position on the chromosome (Fig 3). In contrast, when the pPSV38-NGFP-ParB plasmid was introduced in a Δ*parA* mutant, foci were mispositionned and their interfocal distance was strongly reduced (Fig 3). These results indicate that ParB bound to *parS* is positioned near the 0.2/0.8 relative cell length in a ParA dependent manner, but independently of the location of *parS* on the chromosome.

**Fig 4:**
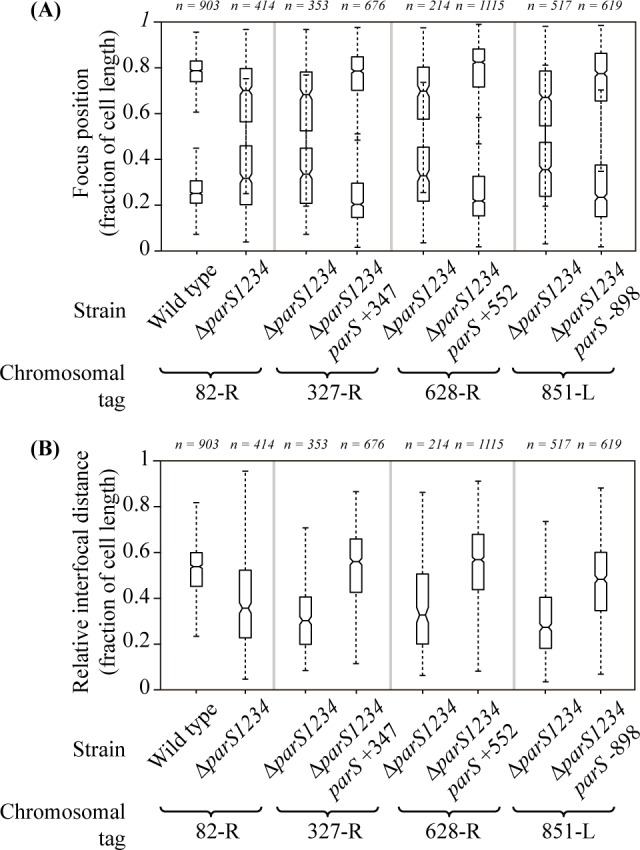
Positioning inside the cells of chromosomal loci located close to an ectopic *parS* site. (A) represents the localization of the 2 foci in cells (randomly oriented) containing two foci, for each genetic background, whereas (B) represents the interfocal distance between these two foci. Boxplot representations are used, indicating the median (horizontal bar), the 25th and the 75^th^ percentile (open box) and the rest of the population except for the outliers (whiskers). Outliers are defined as 1.5×*IQR* or more above the 75^th^ percentile or 1.5×*IQR* or more below the first 25th percentile quartile. Genetic background in which each locus is observed are indicated, as well as the number of cells considered. Positions of the chromosomal tags: 82-R, +82 kb from *oriC* (strains IVGB492 and VLB21); 327-R, +327 kb from *oriC* (strain IVGB478); 628-R, +628 kb from *oriC* (strains VLB23 and VLB140); 851-L, -851 kb from *oriC* (strains IVGB509 and IVGB510).

To determine whether the ParB/*parS* complex positioning at the 0.2/0.8 relative cell length impacts chromosomal loci adjacent to *parS*, we analyzed the localization of chromosomal tags in different strains. We used three different tags, located 327 kb or 628 kb from *oriC* on the right replichore, or 851 kb from *oriC* on the left replichore (327-R, 628-R and 851-L, respectively). We compared their positioning inside the cell in the Δ*parS1234* mutant with their positioning when a *parS* site was located close by (in the Δ*parS1234 parS* +347, Δ*parS1234 parS* +552 and Δ*parS1234 parS* -898 strains, respectively). As a control, we used a chromosomal tag located 82 kb from *oriC* on the right replichore. As previously described, this tag is positioned at the 0.2/0.8 relative cell length in the wild type strain (with an interfocal distance of 0.6 relative cell length), and this positioning is lost in a Δ*parS1234* mutant. Results presented in Fig 4 indicate that all three chromosomal tags are positioned near the 0.2/0.8 relative cell length when a *parS* site is located nearby (at 20 kb, 76 kb and 47 kb from the observed tag, respectively), in contrast to what happen in a Δ*parS1234* mutant. This is surprisingly also the case for the Δ*parS1234 parS* -898 strain, which presents 25% of anucleate cells, a level similar to that of the Δ*parS1234* mutant.

### The *parS* site is the site of force exertion during the segregation process

**Fig 5:**
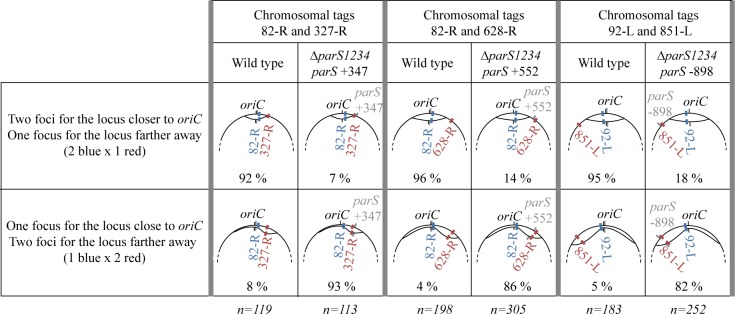
The *parS* site is the site of force exertion of the segregation process. Fractions of cells presenting 3 foci for two chromosomal tags in different genetics background (shown in top) are indicated in the wild type strain, and in a strain with an ectopic *parS*. Numbers of cell considered are shown below. Schematics of the different chromosomal configurations are represented; the position of the *parS* site is indicated in grey, the position of *oriC* in black, and chromosomal loci in blue and red. Strains IVGB292, IVGB168 and IVGB173 were used for the wild type background, as well as strains IVGB478 (Δ*parS1234 parS* +347 background), VLB140 (Δ*parS1234 parS* +552 background) and IVGB510 (Δ*parS1234 parS* -898 background).

We analyzed the impact of the *parS* site chromosomal location on chromosomal loci separation after replication. We used strains with two chromosomal tags allowing to visualize two chromosomal loci in the same cells: one close to *oriC*, and another farther away. We compared the number of foci visible in the wild type strain (in which the four *parS* located between 3 and 15 kb of oriC are assimilated to one *parS*) with the number of foci visible in a strain containing an ectopic *parS*. The rational was that to appear as 2 foci, a chromosomal locus must be replicated and separated upon replication. We used three combinations of chromosomal loci to analyze the impact of three *parS* positions that lead to different amount of anucleate cells: *parS* +347 (less than 5%); *parS* -898 (around 25%); and *parS* +552 (around 10%) (see Fig 2). Complete foci repartitions for each strain are shown in S6 Fig. Fig 5 considers only the cells for which 3 foci are visible, *i*.*e*. cells in which one chromosomal locus was separated after replication but not the other. In the wild type background, the chromosomal locus close to *oriC* was separated first in more than 90% of the 3 foci cells, which is expected considering that it is replicated first and also close to the *parS* sites. Strikingly, in the ectopic *parS* backgrounds, more than 80% of the 3 foci cells contained 1 focus for the locus proximal to *oriC* (replicated first) and 2 foci for the locus located proximal to the *parS* site, (replicated last). This indicate that an active process is required to separate replicated chromosomal loci, and that it originates from the *parS* site, which thus appear to be the site of force exertion of the segregation process.

Altogether, these results show that inserting a *parS* site at an ectopic location induces a repositioning of this *parS* site and of the adjacent chromosomal loci, and that *parS* are the first sequences to be separated after replication. This is strikingly the case for all three tested locations of *parS*, despite the fact that the Δ*parS1234 parS* +347, Δ*parS1234 parS* +552 and Δ*parS1234 parS* -898 strains produce different amount of anucleate cells, as observed upon nucleoid staining. This suggests that the defect in chromosome segregation observed in the Δ*parS1234 parS* -898 strain does not originate from the inability of the ParABS system to position chromosomal loci flanking the *parS* site close to the 0.2/0.8 relative cell length.

### Identification of a “competence zone” for *P*. *aeruginosa parS* site

To better characterize the position effect of the *parS* site, independently of putative local effects due to *parS* insertion, we used a high-throughput approach. We inserted a *parS* site in a *mariner* transposon (modified from the pSC189 vector, [29]), and used it to generate a randomly inserted transposon library in the Δ*parS1234* mutant. A *parS* site inserted at a position for which chromosome segregation occurs correctly (few anucleate cells) is expected to have a fitness advantage. Therefore, although the transposon library would have *parS* inserted at random locations over the genome, the ones having a *parS* in position allowing correct chromosome segregation would be enriched during the propagation. As a control, an insertion library build with a transposon without *parS* was carried out in parallel and insertions sites of these 2 libraries were then identified according to the protocol described in [30] (see Methods for details).

**Fig 6:**
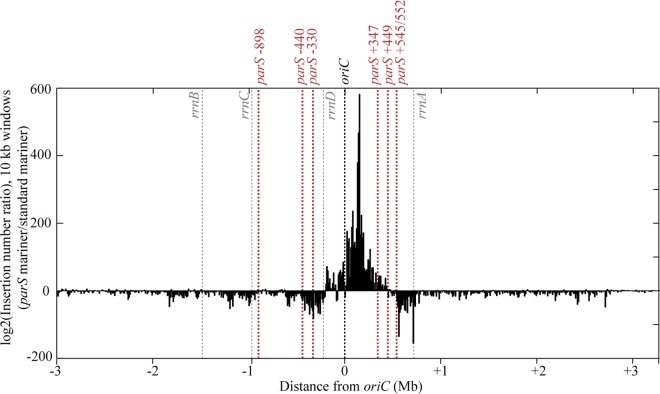
Identification of the “competence zone” of *P*. *aeruginosa parS* site. Log2 of ratio of insertion numbers (normalized to the total number of reads for each experiment) of a *mariner* transposon containing a *parS* site and a standard *mariner* transposon in the Δ*parS1234* mutant, calculated for 10 kb windows, and represented according to the distance from *oriC*. The black dashed line represents the position of *oriC*, the grey dashed lines the position of the ribosomal operons, and the red dashed lines the position of the *parS* sites introduced in the Δ*parS1234* mutant presented in this study (Fig 2).

Ratios between the number of insertions using the two transposons are presented in Fig 6. A clear enrichment of *parS* insertions is observed in the region between approximately -200 kb to +450 kb from *oriC*. It is due to a very strong bias of insertions obtained with the *mariner* transposon containing a *parS* site, but not the control transposon. This indicates that insertion of a *parS* site in this region provides a growth advantage compared to the Δ*parS1234* mutant, whereas an insertion of a *parS* site outside of this region does not. We will refer to this region as the «competence zone» of the *parS* site. In agreement with our previous results, *parS* sites inserted on the left of *oriC* in the Δ*parS1234* (respectively -898, -440 and -330) are located outside of the «competence zone», whereas the *parS* sites inserted on the right of *oriC* are inside (+347 and +449) or at the extreme limit of it (+545 and +552).

**Fig 7:**
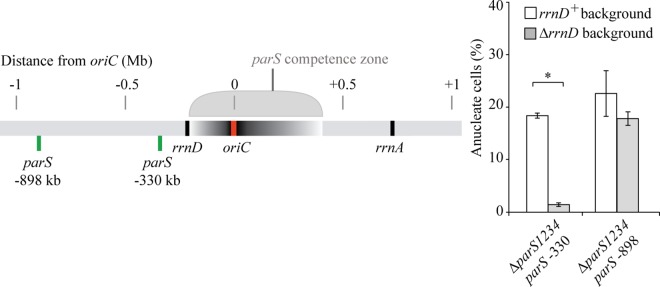
Impact of *rrnD* deletion on the amount of anucleate cells found in liquid culture for strains containing a *parS* site located 330 and 898 kb on the left of *oriC* (Δ*parS1234 parS* -330 (strains IVGB481 and IVGB524) and Δ*parS1234 parS* -898 (strains VLB69 and IVGB526), respectively). A schematic representation of the positioning of *parS* and *rrnD* on the chromosome is shown on the left. Histograms and error bars represent the mean and standard deviation for at least three independent experiments. Significant differences between strains were determined by *t* test. *, *P*<0.001.

Remarkably, we noticed that the «competence zone» is not centered on the *parS* native position, in the vicinity of *oriC*, and that the left border might coincide with a ribosomal operon (located at -220kb on the left of *oriC*). We deleted this ribosomal operon in the Δ*parS1234 parS* -330 and Δ*parS1234 parS* -898 strains (generating strains Δ*parS1234 parS* -330 Δ*rrnD* and Δ*parS1234 parS* -898Δ*rrnD*), and analyzed the efficiency of chromosome segregation in the resulting strains by nucleoid staining. The proportion of anucleate cells in the Δ*parS1234 parS* -330 Δ*rrnD* strain was strongly reduced compared to the Δ*parS1234 parS* -330 strain (2% compared to 20%), indicating that the *rrnD* operon, which is located between *parS* and *oriC*, impairs chromosome segregation (Fig 7). We note however that the proportion of anucleate cells in the Δ*parS1234 parS* -898 Δ*rrnD* strain was close to 20%, indicating that deleting the *rrnD* operon does not restore efficient chromosome segregation in a strain containing a *parS* site located 898 kb on the left of *oriC*. Altogether the observed asymmetry of the competence zone is likely due to the presence of the *rrnD* operon between *oriC* and *parS*.

### The *parS* «competence zone» is determined by the proximity between *parS* and *oriC*

To identify genetic determinants of the *parS* «competence zone», we used the λ derived site-specific recombination system [31] to invert chromosomal fragments and bring specific sequences closer to *parS* sites located outside of the «competence zone». The impact of these programmed chromosome rearrangements on anucleate cell formation was then analyzed by nucleoid staining.

**Fig 8:**
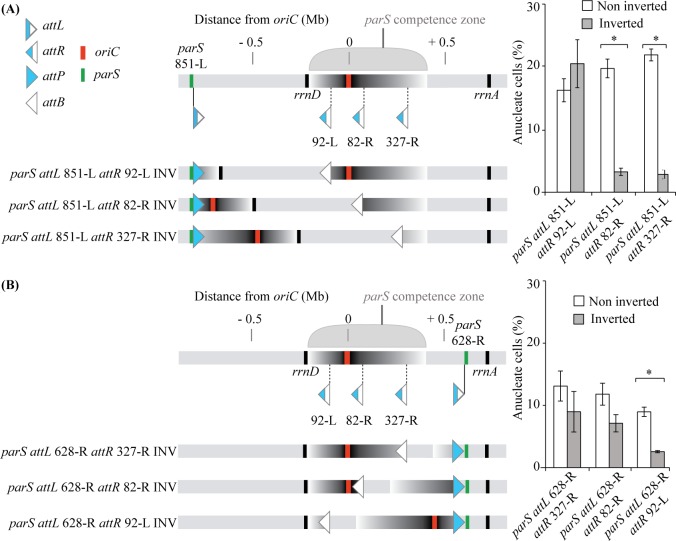
Genetic analysis of the *parS* “competence zone”. Generation of programmed chromosome rearrangements (using the lambda derived recombination system) bringing part of the “Competence zone” closer to a *parS* site located 851 kb on the left of *oriC* **(A)** or 628 kb on the right of *oriC* **(B)**. Schematic representations of the inverted regions, as well as the position on the chromosome of the *parS*, *attL* and *attR* sites, *oriC* and *rrn* operons are shown on the left. Impact of these inversions on the amount of anucleate cells is shown on the right. Histograms and error bars represent the mean and standard deviation for at least three independent experiments. Significant differences between strains were determined by *t* test. *, *P*<0.001. Strains VLB271, VLB272, VLB 273, VLB276, VLB277 and VLB278 were used in (A), and strains IVGB556, IVGB557, IVGB558, VLB333, VLB334 and VLB335 were used in (B).

More specifically, we inserted a *parS* site next to an *attL* site at position 851-L (851 kb on the left of *oriC*, see methods), and *attR* sites at three positions: 92-L (-92 kb from *oriC*), 82-R (+82 kb from *oriC*) or 327-R (+327 kb from *oriC*). Recombination between *attL* and *attR* sites upon Int and Xis action led to the inversion of the chromosomal region between these sites, whereas the position of the *parS* site remains unchanged. The amount of anucleate cells observed in the inverted strains was compared to the one in the parental strains (Fig 8A). In the case of *attR* 92-L, no difference in anucleate cells could be detected whereas with *attR* 82-R and 327-R, a significant decrease was observed in the inverted strains compared to the non-inverted strains. Therefore, when a *parS* is located at position 851-L, efficient chromosome segregation can occur when *oriC* is moved to a closer position (only 83 kb or 328 kb away from *parS*, respectively). It is noteworthy to mention that in these strains, *rrnD* is not located between *parS* and *oriC* (Fig 8A).

The same approach was used to analyze the right side of the «competence zone»: an *attL* site next to a *parS* site was inserted at position 628-R (628 kb on the right of *oriC*), and *attR* sites were inserted at positions 327-R, 82-R or 92-L respectively. Remarkably, a significant decrease in anucleate cells production was only observed in the inverted strain containing the *attR* 92-L, i.e. when *oriC* was translocated 93 kb away from *parS* (Fig 8B).

Altogether, these results demonstrate that genetic determinant(s) of the *parS* «competence zone» is located between -92 kb and +82 kb from *oriC*. This region encompasses a number of essential genes, but its most striking feature is that it contains *oriC* itself. Therefore, we propose that the «competence zone» is defined by the distance between *oriC* and *parS*, and that proximity between these two sequences is critical for efficient chromosome segregation.

## Discussion

### *In vivo* binding of ParB in *P*. *aeruginosa*

Using a ChIP-seq approach, we identified 4 strong ParB binding sites *in vivo*, which allow us to define the TGTTCCACGTGGAACM sequence as the *P*. *aeruginosa parS* site. Although *in vitro* 2 mismatches did not affect ParB binding to this sequence drastically, [21,27], no significant enrichment of such degenerated sequences was detected *in vivo*, either in the wild type strain or in the strain deprived of the 4 *bona fide parS* sites. We also demonstrate that re-positioning a 2 mismatches sequence closer to *oriC* does not allow ParB binding.

Interestingly, we identified nine secondary binding sites for ParB. These sites are less efficiently bound than the *parS* sites, no major “spreading” phenomenon could be observed, and these sites are not involved in chromosome segregation. They might be involved in transcriptional regulation by ParB, and this is of particular interest in the case of the secondary site identified in the promoter region of *dnaA*, a gene encoding the replication initiation protein in bacteria. A link between the ParABS system and the regulation of replication initiation has been characterized in *B*. *subtilis* and *V*. *cholerae* chromosome I. However, it most probably occurs post-transcriptionally, through the interaction of ParA with DnaA, and not at the transcriptional level [32]. The mechanism of ParB binding to these secondary sites and their biological significance remain to be characterized.

It is noteworthy to mention that the enrichment profiles surrounding the *parS* sites encompasses more than twenty kb and is compatible with the presence of ParB molecules involved either in a “spreading and bridging” mechanism [11], or a “nucleation and caging” mechanism [12] of binding. This is consistent with the fact that it was previously shown that *P*. *aeruginosa* ParB was able to bridge DNA *in vitro* [10].

### Functioning of the ParABS system of *P*. *aeruginosa*

We show that in our growth conditions, one *parS* site is sufficient to promote proper chromosome segregation, as was described in the study from Jecz and colleagues [27]. However, the amount of anucleate cells in a strain deprived of *parS* sites differs between the 2 studies (more than 20% in our case, in contrast to 2–3% in the work of Jecz and colleagues [27]). We use a PAO1 isolate that presents differences from the sequenced isolate [33], whereas they use the PAO1161 strain, which is a derivative of the sequenced PAO1 [19], which might account for this difference. In this study, we also demonstrate that the location of the *parS* site on the chromosome is critical for chromosome segregation (as assessed by measuring growth rate and anucleate cells in liquid cultures), and identify what we called a «competence zone» for *parS*, which ranges roughly from 200 kb on the left of *oriC* (where it is limited by *rrnD*) to 450 kb on its right. Strikingly, we were unable to construct and propagate strains containing a *parS* site located at either 1.5 Mb or 1 Mb on the right of *oriC*, suggesting that the introduction of a *parS* site far from *oriC* can be more detrimental for growth than the absence of *parS* site.

We also determined that ParB binds to *parS* independently of ParA and of the *parS* location on the chromosome, and that ParB bound to *parS* is positioned to the 0.2/0.8 relative cell length. This localization depends on ParA, but not on the *parS* location on the chromosome. These results suggest a repositioning of the chromosomal region containing the *parS* site due to the ParB and ParA proteins, which could be linked to an “anchorage process” through ParA. Indeed, a repositioning of chromosomal loci containing ectopic *parS* has been previously described in *Caulobacter crescentus* [34] and *V*. *cholerae* chromosome I [35], two bacteria that possess specific proteins allowing the anchorage of the *parS* sites to the cell pole [36–38]. Interestingly, no anchorage mechanism to cellular positions other than cell poles has been described so far. In contrast, introduction of *parS* arrays at different locations on the *B*. *subtilis* chromosome does not induce a repositioning of the chromosomal loci in which they are inserted [39], and no anchorage process was described in this bacterium. Subpolar positioning of *parS* sites have also been described in *Myxococcus xanthus*, however the impact of their displacement on chromosomal loci positioning has not been assessed yet [40].

### Proximity between the *parS* site and *oriC* define a «competence zone» for segregation

By engineering chromosome rearrangements using a lambda-based recombination system, we demonstrate that the «competence zone» is linked to the distance between *parS* and *oriC*. Strikingly, this is in perfect agreement with the observation that *parS* sites are found near *oriC* in the vast majority of bacterial species [6], although a functional link was not demonstrated previously. In contrast, it was reported that *parS* sites could be moved 650 kb away from *oriC* in the 3 Mb long *V*. *cholerae* chromosome I without impacting the efficiency of chromosome segregation [35]. Similarly, no defect was described when *parS* sites where displaced 400 kb away of *oriC* in the 4 Mb long *C*. *crescentus* chromosome [41]. To our knowledge, the only previous hint of functional coupling between *parS* site and *oriC* has been described in *B*. *subtilis*. In this bacterium, the chromosome segregation defect observed when *parS* sites were inserted near the terminus of replication was mainly due to the recruitment of the SMC condensin away from *oriC*. SMC is the prominent factor involved in *B*. *subtilis* chromosome segregation [14]. In *P*. *aeruginosa*, a Δ*smc* mutant presents only a slight defect in chromosome segregation [25]. However, we were not able to delete *smc* in the Δ*parS1234 parS* -898 strain, and deletion of *smc* in the Δ*parS1234 parS* -330 Δ*rrnD* strain leads to an increase in anucleate cells (from approximately 1% to approximately 7%), which indicates that unlike the situation described in *B*. *subtilis*, the segregation defect observed when the *parS* site is distant from *oriC* is not linked to SMC function.

In this study, we establish that the *parS* site is the site of force exertion of the segregation process, (as described in *C*. *crescentus* [41]); indeed, chromosomal loci close to the *parS* site are the first to be separated after replication. Moreover, they reach the 0.2/0.8 relative cell length upon segregation, independently of *parS* location on the chromosome. Strikingly, this is also the case in the Δ*parS1234 parS* -898 strain for which more than 20% of anucleate cells are observed. This suggests that chromosome segregation in this strain does not originate from a defect of positioning of the chromosomal loci surrounding the *parS* site, or from a defect in the “segregation force” itself. Our demonstration that bringing *oriC* closer to the *parS* site restores proper chromosome segregation indicate that the timing of separation of the chromosomal loci surrounding *oriC* after replication initiation might be critical, and that it is normally determined by the distance between *oriC* and *parS*. When this distance is too large, or when a *rrn* operon is located between *parS* and *oriC*, the “segregation force” is still applied but the timing of separation of *oriC* is lost, and this is detrimental for the segregation process. Interestingly, two studies in *B*. *subtilis* also indicate that a failure in separating newly replicated origins is detrimental for the segregation process [42,43]. However, the specific problems arising from the delayed separation of the replicated *oriC* remains to be characterized.

It was previously shown that *rrn* operon can interfere with chromosome organization and form conformational barriers [17,44]. It was proposed that these barriers serve as flexible tethers creating a spatial gap between domains, which might be consistent with our results: when such a barrier is inserted between *parS* and *oriC* in *P*. *aeruginosa*, the timing of separation of replicated *oriC* is lost, even if the *parS* site is not too far from *oriC*. An alternative explanation might be that *rrn* operons are transcribed altogether, in a nucleolus-like structure, which would interfere with the segregation process originating from *parS*, and impair once again the timing of *oriC* separation. However, even if clusters of RNA polymerase have been observed [45], the existence of nucleolus-like structure in bacteria remains to be proven. The presence of a ribosomal operon between *oriC* and the *parS* site is not always detrimental to chromosome segregation. Indeed, this is the case for *Vibrio cholera* chromosome I (rDNA found at + 53 kb from *oriC* when the 3 *parS* sites are at + 63,+ 66 and + 69 kb from *oriC*), and several ribosomal operons alternate with *parS* sites on the *oriC* proximal part of the right chromosomal arm in *B*. *subtilis*. In both cases however, the impact of the ParABS system impairment on chromosome segregation is marginal.

Overall, this study demonstrates a functional link between *oriC* and *parS* in *P*. *aeruginosa*. It suggests that the timing of separation of the chromosomal loci surrounding *oriC* after replication is critical, and that it could be an important role of the ParABS system to keep this timing right, which would explain the proximity of *parS* and *oriC* in most bacterial species.

## Materials and Methods

### Strains and media

*P*. *aeruginosa* strain PAO1 was initially provided by Arne Rietsch (Case Western Reserve University). This PAO1 isolate does not present the inversion described for the sequenced PAO1-UW subclone resulting from homologous recombination between the *rrnA* and *rrnB* loci, which are orientated in opposite directions and separated by 2.2 Mbp [19]. It also contains the 12 kb insertion and 1006 bp deletion described in [33]. *Escherichia coli* DH5α (Invitrogen) and DH5α λ*pir* was used as the recipient strain for all plasmid constructions, whereas *E*. *coli* strains β2163 [46] and MPFpir [47] were used to mate plasmids into *P*. *aeruginosa*. Details of plasmid and strain constructions are provided in S1 Text and S3 Table.

For growth rate and anucleate cells analysis, overnight cultures grown in Lysogeny broth (LB) at 37°C were diluted 300 times in Minimal Medium A (Miller 1992) supplemented with 0.12% casamino acids and 0.5% glucose, and strains were grown at 30°C until they reach an OD of approximately 0.15. For fluorescent microscopy analysis of chromosomal loci and for NGFP-ParB localization, Minimal Medium A supplemented with 0.25% citrate was used. IPTG was added to growth medium at 0.5 mM for observation of chromosomal tags and 0.1 mM for observation of NGFP-ParB until they reach an OD of approximately 0.1.

### Fluorescent microscopy analysis

Cells were grown until OD600 0.1, and fixed with an equal volume of a 1×PBS solution containing 5% paraformaldehyde and 0.06% glutaraldehyde. After overnight incubation at 4°C, the cells were washed twice in PBS and then incubated in a solution of 1 μg ml^−1^ HOESCHT 33258 (Thermofisher). After 20 min incubation, the cells were washed in 1×PBS, spread out on agarose pads and observed immediately using a Leica DM6000 microscope, a coolsnap HQ CCD camera (Roper) and Metamorph software.

Chromosomal loci and NGFP-ParB were observed when cultures reached an OD600 between 0.05 and 0.1. Cells were then spread out on agarose pads and observed immediately using a Leica DM6000 microscope, a coolsnap HQ CCD camera (Roper) and Metamorph software. Image analysis was performed using the MATLAB-based software MicrobeTracker Suite [48]. Briefly, The MicrobeTracker program was used to identify cell outlines, and SpotFinderZ to detect fluorescent spots inside the cells. Spots and cell outlines were then manually validated using homemade matlab functions [35]. Spot numbers and positions were then analyzed according to cell length.

### Chromatin Immunoprecipitation (ChIP)

Formaldehyde was added to 150 ml of culture (Minimal Medium A supplemented with 0.25% citrate, OD600 approximately 0.1) to a final concentration of 1% and samples were incubated at room temperature for 30 min. To quench cross-linking reaction, glycine was added to a final concentration of 125 mM and followed by a 10 min incubation at room temperature. Cell pellets were washed three times with 10ml of PBS and then resuspended in 1 ml of lysis buffer (50 mM Tris-HCl pH 7.4; 150 mM NaCl; 1 mM EDTA; Triton X-100 1%; and Roche Protease Inhibitor Cocktail). Chromosomal DNA was sheared by sonication to an average size of 0.5–1 kb. After the removal of cell debris by centrifugation, 50 μl of each sample was removed to serve as an input control. The remaining samples were added to 50 μl of ANTI-FLAG M2 affinity resin (Sigma A2220) previously washed twice with TBS and twice with lysis buffer. After incubation at 4°C overnight, beads were pelleted and washed twice with TBS Tween 0.5% and three times with TBS. Elution was performed using the 3X FLAG peptide (Sigma F4799), as recommended. The recovered supernatants were placed at 65°C overnight to reverse the cross-links. The input samples were also incubated at 65°C overnight after the addition of 200 μl of TBS.

### ChIP-seq analyses

Library preparation and sequencing were performed by the IMAGIF facility (I2BC, Gif sur Yvette). Sequences were aligned against the reconstituted genome of our PAO1 strain (available on request). The sequencing results were analyzed as described in [49]. Briefly, the number of reads for the input and IP data was smoothed over a 200 bp window (the estimated size of the fractionated DNA), normalized to the total number of reads, and enrichments fold were calculated for each base as the ratio of the number of reads in the IP fraction and the number of reads in the input fraction. Peak calling was done using Matlab functions. Regions presenting a tenfold enrichment were selected, except in the Δ*par123* strain, for which an enrichment of 15 was preferred. The rational was to select for the approximatively 10 most abundant regions. Considering that only the 4 *parS* sites are involved in chromosome segregation, further investigation of smaller peaks was not undertaken. Results are presented in S1 Table. Sequencing data are available from the GEO database via accession number GSE87409 (http://www.ncbi.nlm.nih.gov/geo/).

### Tn-seq experiments and analysis

The pSC189 vector, described in [29], was modified for use in *P*. *aeruginosa*. The *aacC1* gene from pEXG2 [50] was PCR amplified and cloned on a *Xho*I/*Nco*I fragment into the pSC189 vector, giving rise to pSC189Gm. Then, 129 pb containing the *parS3* site were PCR amplified from PAO1 chromosome and cloned on a *Xho*I fragment into the pSC189Gm digested with *Sal*I, giving rise to pSC189Gm-*parS*. pSC189Gm and pSC189Gm-parS were then tranformed into strain MFPpir for further mating into the Δ*parS1234* mutant.

Mutant libraries were generated according to [51]. Briefly, donor strains (MFPpir containing whether pSC189Gm or pSC189Gm-*parS*) and recipient strain (Δ*par1234*) were scraped from overnight plates grown at 37°C and 42°C, respectively. Optical densities were adjusted to 40 for the donor strain and 20 for the recipient strain. Equivalent volumes were mixed, 50 microliter spotted on dried LB plates supplemented with 0.3 mM of DAP (Sigma D1377), and incubated overnight for transposition and selection. Mating mixtures were scraped and plated on Pseudomonas Isolation Agar (Sigma 17208) containing 60 μg mL^-1^ of Gentamicin (Sigma G1397). Sixty matings were done, giving rise to approximately 80,000 colonies per mutant library. Colonies were scraped from plates, washed once in LB and froze in 10% DMSO at -80°C in 5 aliquots.

Genomic DNA was prepared from one aliquot using the GenElute™ Bacterial Genomic DNA Kit from Sigma, and sequencing libraries were prepared as described in [30]. Sequencing was performed at the IMAGIF facility (I2BC, Gif sur Yvette). Sequences were aligned against the reconstituted genome of our PAO1 strain to determine the insertion locations of transposons. (Raw data are available from the SRA database (https://trace.ncbi.nlm.nih.gov/Traces/sra/) under accession number SRP090425). Ratio between numbers of insertion of the pSC189Gm-*parS* and pSC189Gm were calculated, log2 calculated to improve the representation of the data, and results were binned over 10 kb.

## Supporting Information

S1 Fig Construction of a functional ParB-3xFLAG fusion. (A) Schematic representation of the 3xFLAG tag integration vector and its use to construct the PAO1 ParB-3xFLAG strain that synthesizes the ParB protein with a 3xFLAG tag (ParB-3xFLAG) at native levels. (B) Generation times and amounts of anucleate cells of the PAO1 wild type strain and the PAO1 ParB-3xFLAG derivative (Strain IVGB379) (C) Positioning of chromosomal loci located in the Ori region (82-R, left panels) and in the Ter region (3,028-R, right panels) in the PAO1 ParB-3xFLAG and (D) in the PAO1 strains grown in minimal medium supplemented with citrate (respectively strains IVGB396, IVGB397 and IVGB123). The position of the foci in cells containing 1 (upper panels) or 2 (bottom panels) foci are presented. Both loci were visualized individually. Cells for the Ori locus were randomly oriented, whereas cells for the Ter locus were arbitrarily oriented to have foci closer to the 0 pole.
(TIF)

S2 Fig Comparison of ChIP-seq results for ParB-3xFLAG with ChIP-seq results for untagged strain–Identification of secondary peaks specific of ParB binding on *P*. *aeruginosa* chromosome. (A) Each panel represents a zoom of Fig 1A, of each 10 kb region containing a secondary peak identified in the wild type background. Letters refer to Fig 1 and S1 Table. The red dotted line indicates the 10 fold enrichment limit. (B) Chromatin Immunoprecipitation using an anti-3xFLAG antibody in the PAO1 ParB-3xFLAG strain (Strain IVGB379) or (C) in the PAO1 strain. Enrichment folds between the immunoprecipitated (IP) and the input (IN) fractions are represented for each base of the genome, according to its distance from *oriC*, and ratio of these enrichment folds are represented in (D). The insets represent a zoom of the region containing the four *parS* sites bound by ParB. Dashed vertical lines represent the position of the proposed *parS* sites from [21]. Red italicized letters indicated ParB accessory binding sites, and blue italicized letters represents the promoter region of *dnaA*. Grey stars indicate peaks that are less prominent but still found in the different genetic backgrounds. The red dotted lines indicate the significant enrichment.
(TIF)

S3 Fig Representative growth curves for some of the strains presented in this study. OD600 was measured during growth in Minimal Medium supplemented with Glucose and Casamino Acids, and plotted in logarithmic scale according to time. Growth curves for strains PAO1, PAO1 ParB-3xFLAG (IVGB379), Δ*parS123* (IVGB469), Δ*parS1234* (VLB1), Δ*parS1234 parS2* +6.5 (VLB63), Δ*parS1234 parS* +347 (VLB66) and Δ*parS1234 parS* -440 (VLB70) are represented.
(TIF)

S4 Fig Impact of the deletion of the *parS* sites bound by ParB *in vivo* upon chromosome positioning inside the cell. Positioning of chromosomal loci located in the Ori region (82-R, left panels) and in the Ter region (2,957-R, right panels) in the wild type PAO1 strain **(A)** the Δ*parS123* mutant **(B)** and the Δ*parS1234* mutant **(C)** grown in minimal medium supplemented with citrate. The position of the foci in cells containing 1 (upper panels) or 2 (bottom panels) foci are presented. Both loci were visualized in the same cells (using strains IVGB123, VLB13 and VLB21 respectively), which were oriented relative to the Ter locus position (the pole closest to this locus was assumed to be the new pole of the cell). Representative images are shown on the right. The Ori locus is represented in green, and the Ter one in red.
(TIF)

S5 Fig ParB binding to *parS* in the presence of ParA induces its positioning to the 0.2/0.8 relative cell length. The pPSV38-NGFP-ParB plasmid was introduced in different strains, and NGFP-ParB localization was observed in cells grown in minimal medium supplemented with citrate. Foci numbers for each strain are indicated in S2 Table. (A) represents the localization of the 2 foci in cells (randomly oriented) containing two foci, for each genetic background, whereas (B) represents the distance between the two foci. Strains IVGB469 (Δ*parS123*), VLB66 (Δ*parS1234 parS* +347), IVGB480 (Δ*parS1234 parS* +552), VLB69 (Δ*parS1234 parS* -898) and IVGB317 (Δ*parA*) were used.
(TIF)

S6 Fig Foci number repartitions in strains containing chromosomal tags allowing the visualization of two chromosomal loci (82-R and 327-R (left), 82-R and 628-R (middle) and 92-L and 851-L (right)). In each case, these 2 foci were observed in the wild type strain, and in a strain with an ectopic *parS* (Δ*parS1234 parS* +327 (left), Δ*parS1234 parS* +552 (middle) and Δ*parS1234 parS* -898 (right)). Standard numbers indicate the percentage of each category among cells containing foci (the percentage of cells with no focus is indicated below). Numbers of cell considered are indicated. Schematics of the different chromosomal loci observed are represented above each column; the position of the displaced *parS* site is indicated in grey (in the wild type strain, *parS* sites are within 20 kb from *oriC*). Strains IVGB292, IVGB168 and IVGB173 were used for the wild type background, as well as strains IVGB478 (Δ*parS1234 parS* +347 background), VLB140 (Δ*parS1234 parS* +552 background) and IVGB510 (Δ*parS1234 parS* -898 background).
(TIF)

S1 Table Chromatin Immunoprecipitation of ParB-3xFLAG. Precise coordinates of each peak identified after the peak calling procedure, in each genetic background. Corresponding coordinates on the sequenced PAO1 strain from (Stover et al., 2000) are also indicated for clarity purpose. Grey area indicates the *parS* encompassing region of enrichment. The blue area refers to *dnaA* peak. Italicized letters and grey stars refer to peak numbers as indicated in Figs 1 and S2.
(DOCX)

S2 Table Foci number repartition in pPSV38-NGFP-ParB containing strains. % indicates the percentage of each category among the whole population of cells, whereas italicized numbers between brackets indicate the percentage of each category among cells containing foci. Numbers of cells considered are indicated below.
(DOCX)

S3 Table Strains used in this study. (DOCX)

S4 Table Oligos used in this study. (DOCX)

S1 Text Supporting Materials and Methods. (DOCX)
