# Supplementary figures and images for: Regional Control of Chromosome Segregation in Pseudomonas aeruginosa
Source: PLoS Genet. 2016 Nov 7;12(11):e1006428. doi: 10.1371/journal.pgen.1006428 (PMC5098823; doi:10.1371/journal.pgen.1006428)

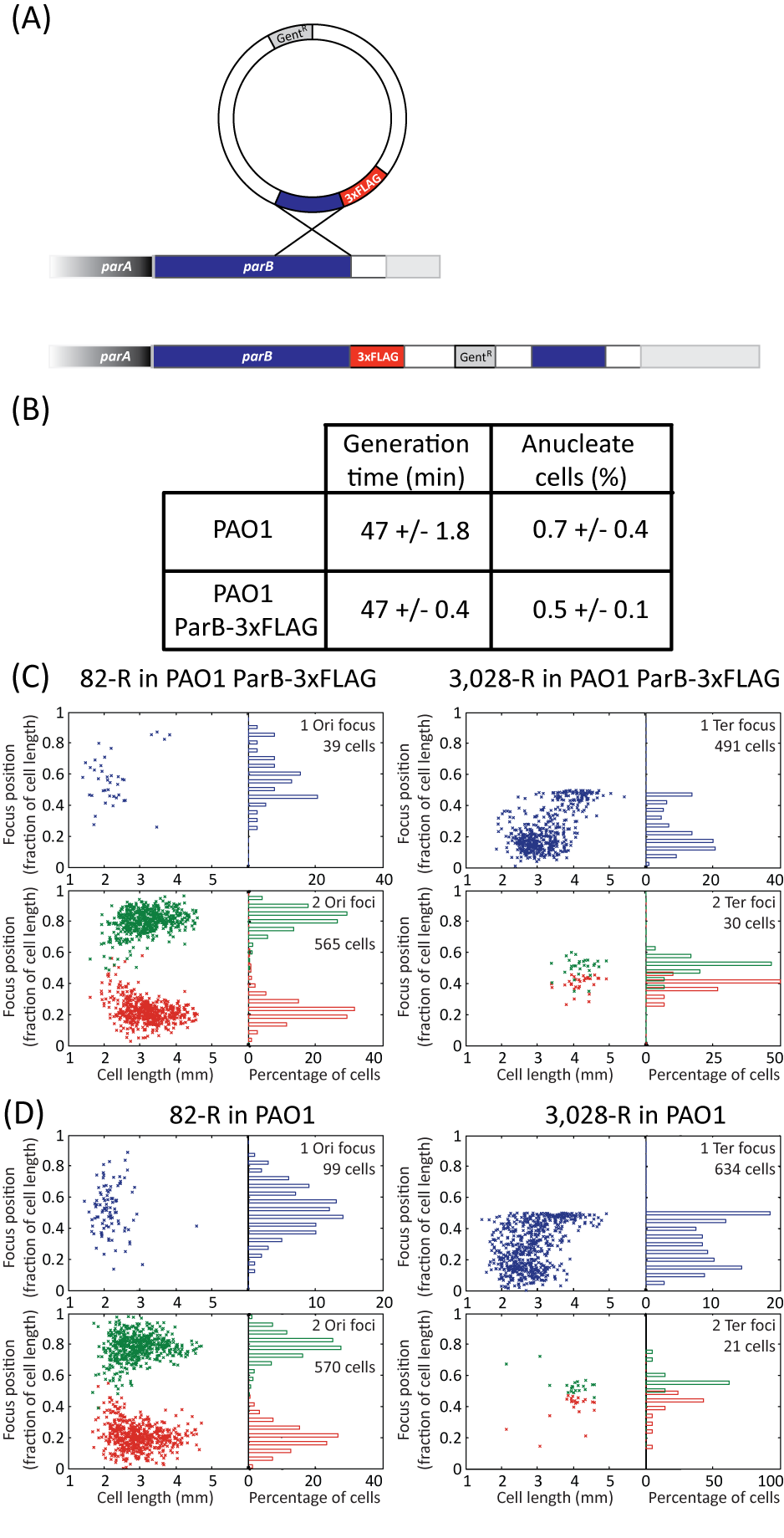

Supplement: S1 Fig — (A) Schematic representation of the 3xFLAG tag integration vector and its use to construct the PAO1 ParB-3xFLAG strain that synthesizes the ParB protein with a 3xFLAG tag (ParB-3xFLAG) at native levels. (B) Generation times and amounts of anucleate cells of the PAO1 wild type strain and the PAO1 ParB-3xFLAG derivative (Strain IVGB379) (C) Positioning of chromosomal loci located in the Ori region (82-R, left panels) and in the Ter region (3,028-R, right panels) in the PAO1 ParB-3xFLAG and (D) in the PAO1 strains grown in minimal medium supplemented with citrate (respectively strains IVGB396, IVGB397 and IVGB123). The position of the foci in cells containing 1 (upper panels) or 2 (bottom panels) foci are presented. Both loci were visualized individually. Cells for the Ori locus were randomly oriented, whereas cells for the Ter locus were arbitrarily oriented to have foci closer to the 0 pole. (TIF) [file pgen.1006428.s001.tif]

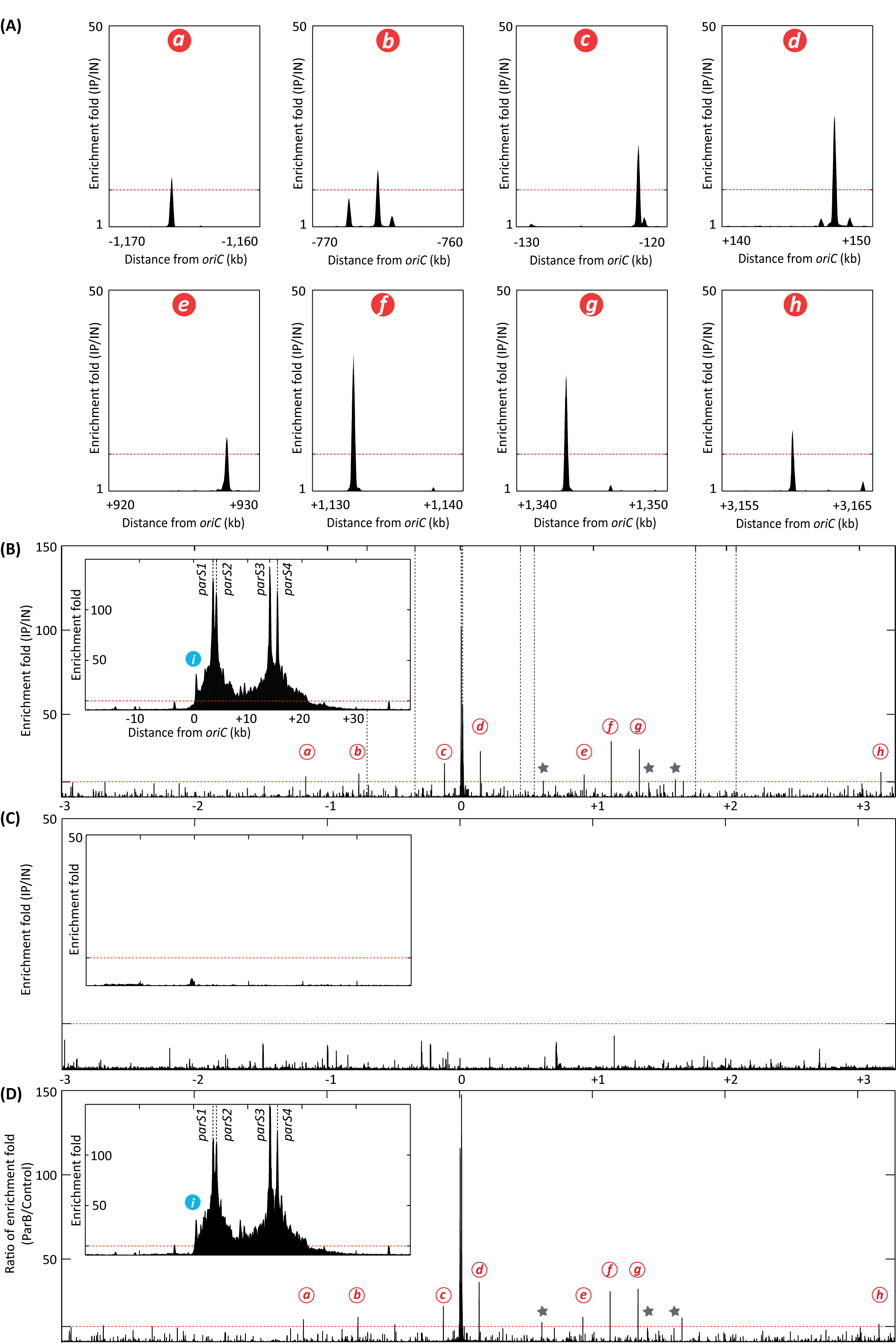

Supplement: S2 Fig — (A) Each panel represents a zoom of Fig 1A, of each 10 kb region containing a secondary peak identified in the wild type background. Letters refer to Fig 1 and S1 Table. The red dotted line indicates the 10 fold enrichment limit. (B) Chromatin Immunoprecipitation using an anti-3xFLAG antibody in the PAO1 ParB-3xFLAG strain (Strain IVGB379) or (C) in the PAO1 strain. Enrichment folds between the immunoprecipitated (IP) and the input (IN) fractions are represented for each base of the genome, according to its distance from oriC, and ratio of these enrichment folds are represented in (D). The insets represent a zoom of the region containing the four parS sites bound by ParB. Dashed vertical lines represent the position of the proposed parS sites from [21]. Red italicized letters indicated ParB accessory binding sites, and blue italicized letters represents the promoter region of dnaA. Grey stars indicate peaks that are less prominent but still found in the different genetic backgrounds. The red dotted lines indicate the significant enrichment. (TIF) [file pgen.1006428.s002.tif]

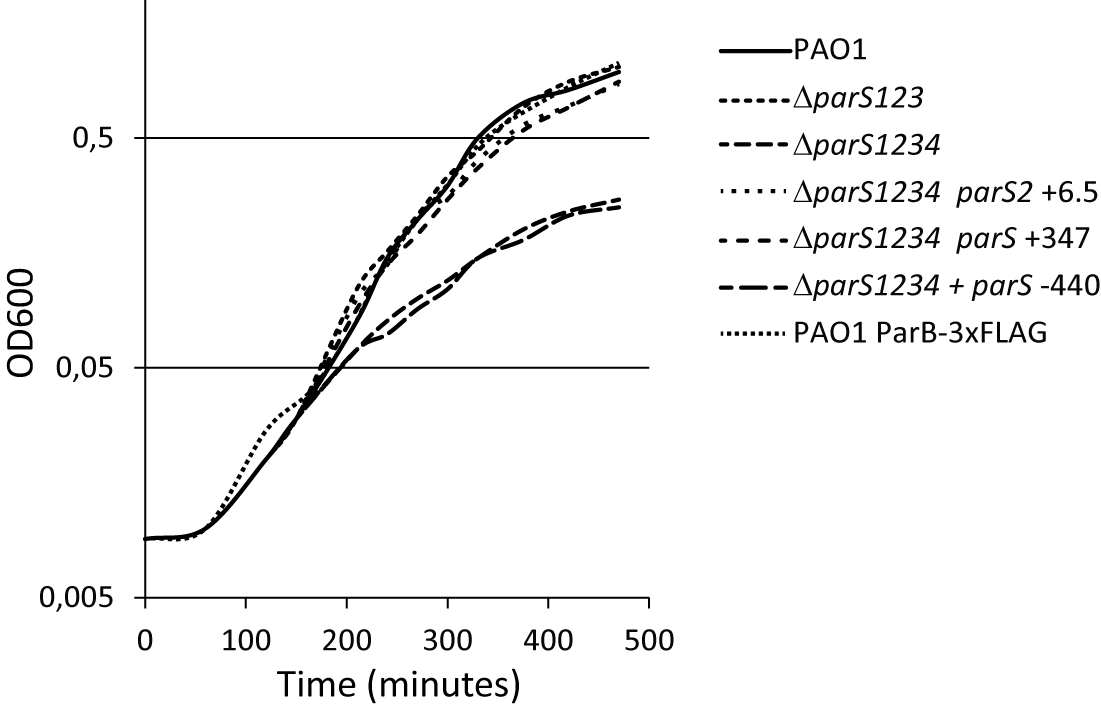

Supplement: S3 Fig — OD600 was measured during growth in Minimal Medium supplemented with Glucose and Casamino Acids, and plotted in logarithmic scale according to time. Growth curves for strains PAO1, PAO1 ParB-3xFLAG (IVGB379), ΔparS123 (IVGB469), ΔparS1234 (VLB1), ΔparS1234 parS2 +6.5 (VLB63), ΔparS1234 parS +347 (VLB66) and ΔparS1234 parS -440 (VLB70) are represented. (TIF) [file pgen.1006428.s003.tif]

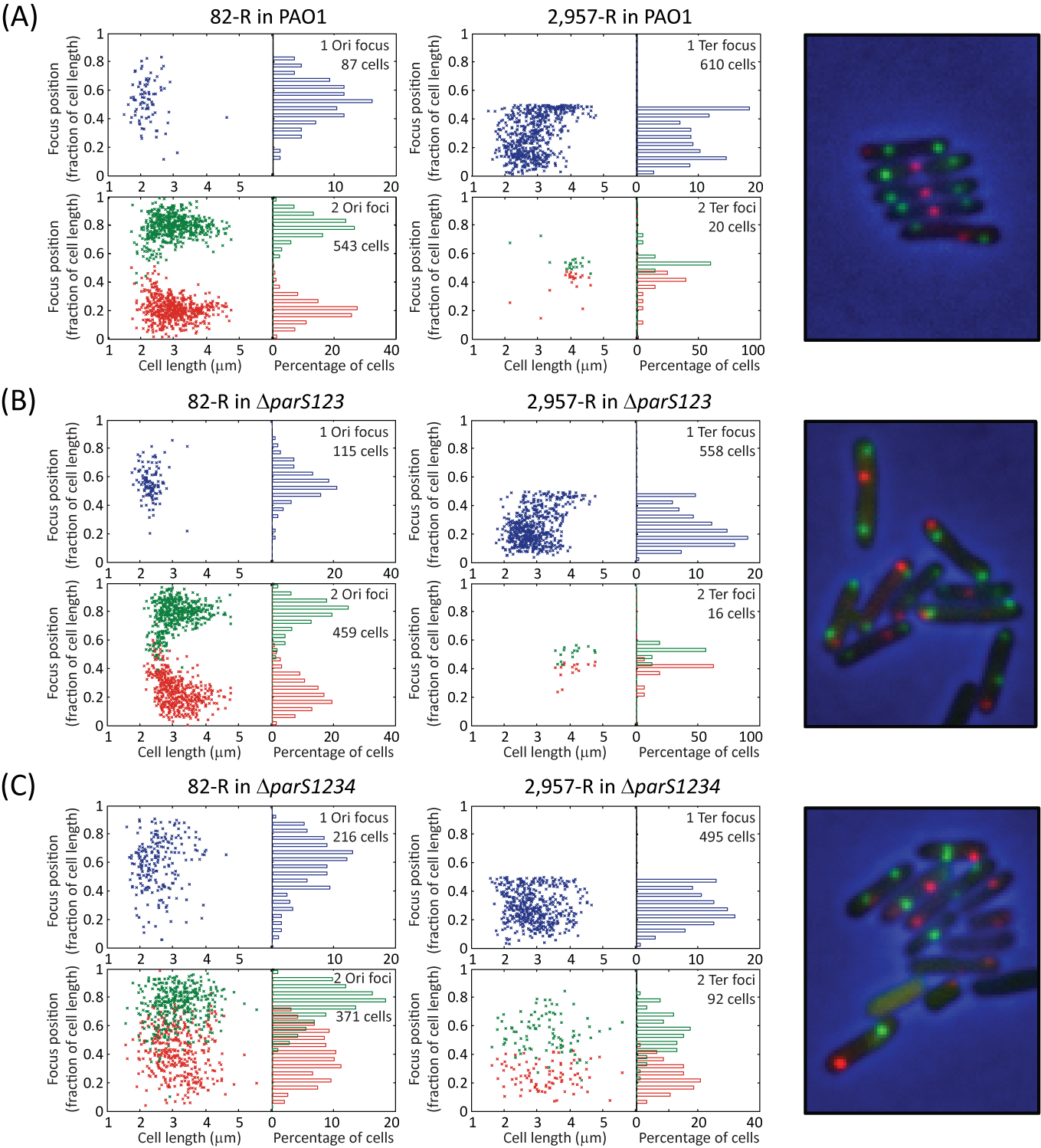

Supplement: S4 Fig — Positioning of chromosomal loci located in the Ori region (82-R, left panels) and in the Ter region (2,957-R, right panels) in the wild type PAO1 strain (A) the ΔparS123 mutant (B) and the ΔparS1234 mutant (C) grown in minimal medium supplemented with citrate. The position of the foci in cells containing 1 (upper panels) or 2 (bottom panels) foci are presented. Both loci were visualized in the same cells (using strains IVGB123, VLB13 and VLB21 respectively), which were oriented relative to the Ter locus position (the pole closest to this locus was assumed to be the new pole of the cell). Representative images are shown on the right. The Ori locus is represented in green, and the Ter one in red. (TIF) [file pgen.1006428.s004.tif]

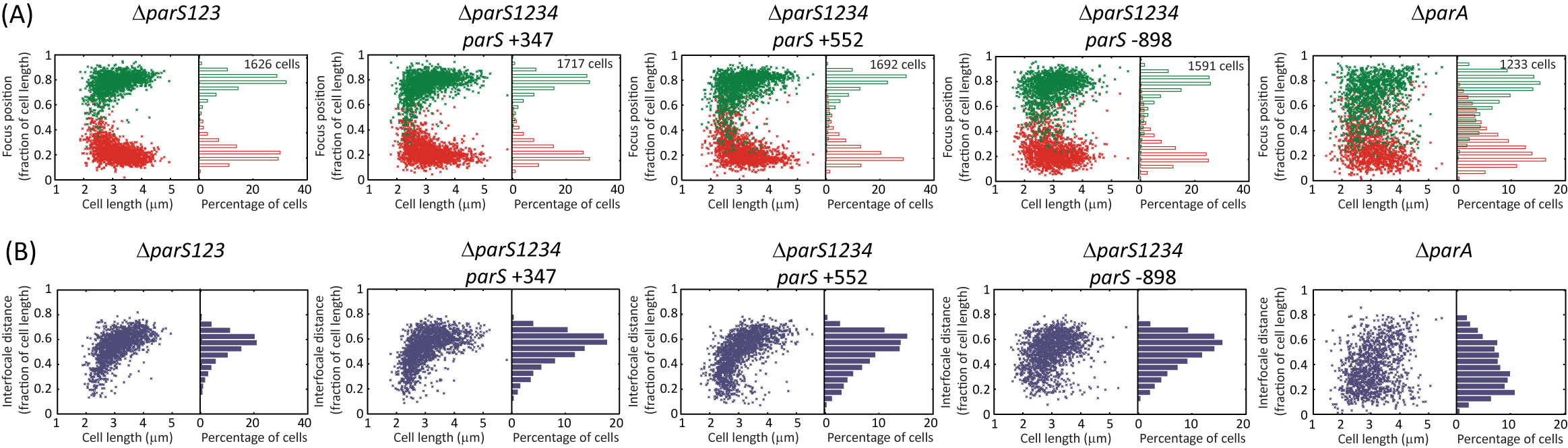

Supplement: S5 Fig — The pPSV38-NGFP-ParB plasmid was introduced in different strains, and NGFP-ParB localization was observed in cells grown in minimal medium supplemented with citrate. Foci numbers for each strain are indicated in S2 Table. (A) represents the localization of the 2 foci in cells (randomly oriented) containing two foci, for each genetic background, whereas (B) represents the distance between the two foci. Strains IVGB469 (ΔparS123), VLB66 (ΔparS1234 parS +347), IVGB480 (ΔparS1234 parS +552), VLB69 (ΔparS1234 parS -898) and IVGB317 (ΔparA) were used. (TIF) [file pgen.1006428.s005.tif]

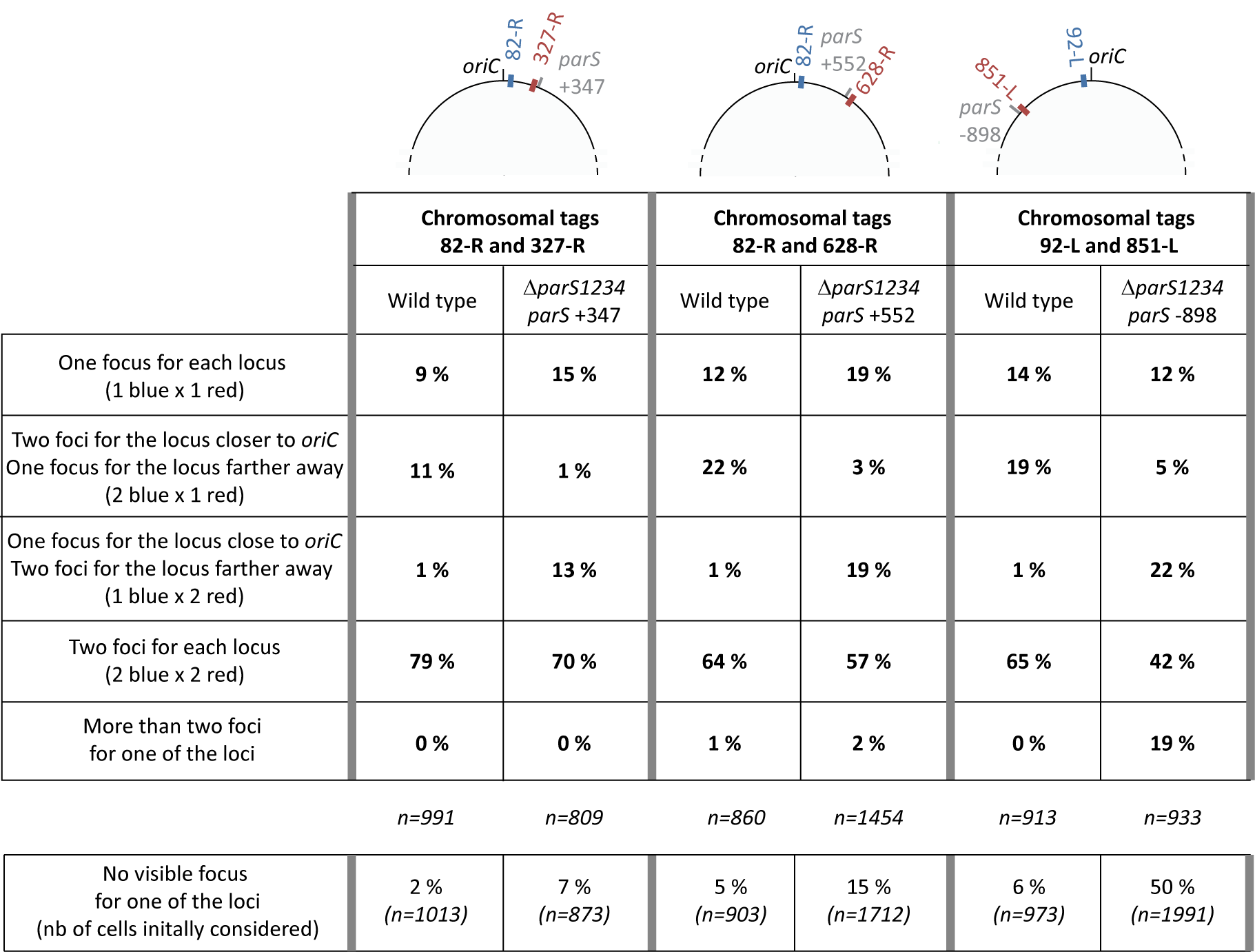

Supplement: S6 Fig — In each case, these 2 foci were observed in the wild type strain, and in a strain with an ectopic parS (ΔparS1234 parS +327 (left), ΔparS1234 parS +552 (middle) and ΔparS1234 parS -898 (right)). Standard numbers indicate the percentage of each category among cells containing foci (the percentage of cells with no focus is indicated below). Numbers of cell considered are indicated. Schematics of the different chromosomal loci observed are represented above each column; the position of the displaced parS site is indicated in grey (in the wild type strain, parS sites are within 20 kb from oriC). Strains IVGB292, IVGB168 and IVGB173 were used for the wild type background, as well as strains IVGB478 (ΔparS1234 parS +347 background), VLB140 (ΔparS1234 parS +552 background) and IVGB510 (ΔparS1234 parS -898 background). (TIF) [file pgen.1006428.s006.tif]
