# Supplementary material for: Regional Control of Chromosome Segregation in Pseudomonas aeruginosa
Source: PLoS Genet. 2016 Nov 7;12(11):e1006428. doi: 10.1371/journal.pgen.1006428 (PMC5098823; doi:10.1371/journal.pgen.1006428)
Supplement: S1 Table — Precise coordinates of each peak identified after the peak calling procedure, in each genetic background. Corresponding coordinates on the sequenced PAO1 strain from (Stover et al., 2000) are also indicated for clarity purpose. Grey area indicates the parS encompassing region of enrichment. The blue area refers to dnaA peak. Italicized letters and grey stars refer to peak numbers as indicated in Figs 1 and S2. (DOCX) [file pgen.1006428.s007.docx]

**Table S1 :** **Chromatin Immunoprecipitation of ParB-3xFLAG.** Precise coordinates of each peak identified after the peak calling procedure, in each genetic background. Corresponding coordinates on the sequenced PAO1 strain from (Stover et al., 2000) are also indicated for clarity purpose. Grey area indicates the *parS* encompassing region of enrichment. The blue area refers to *dnaA* peak. Italicized letters and grey stars refer to peak numbers as indicated in Fig. 1 and S2.* indicates peaks overlapping intergenic regions.

|  | **PAO1 ParB-3xFLAG** | | | | | |  |  | **Δ*parS123* ParB-3xFLAG** | | | |  |  | **Δ*parS1234* ParB-3xFLAG** | | | |
| --- | --- | --- | --- | --- | --- | --- | --- | --- | --- | --- | --- | --- | --- | --- | --- | --- | --- | --- |
| Peak number (from Figure 1) | Start (distance from *oriC*, bp) | End (distance from *oriC*, bp) | Size  (bp) | Enrichment fold | Start (genome from {Stover, 2000 #48}) | End (genome from {Stover, 2000 #48}) |  | Peak number (from Figure 1) | Start (distance from *oriC*, bp) | End (distance from *oriC*, bp) | Size  (bp) | Enrichment fold |  | Peak number (from Figure 1) | Start (distance from *oriC*, bp) | End (distance from *oriC*, bp) | Size  (bp) | Enrichment fold |
| *a* | -1,176,965 | -1,176,848 | 117 | 12 | 5,098,499 | 5,098,616 |  | *a* | -1,176,968 | -1,176,855 | 113 | 23 |  | *a* | -1,176,929 | -1,176,887 | 42 | 23 |
| *b** | -765,758 | -765,590 | 168 | 13 | 5,498,646 | 5,498,814 |  | *b** | -765,761 | -765,598 | 163 | 24 |  | *b** | -765,723 | -765,621 | 102 | 24 |
| *c* | -122,017 | -121,790 | 227 | 16 | 6,142,387 | 6,142,614 |  | *c* | -121,997 | -121,787 | 210 | 30 |  | *c* | -122,005 | -121,793 | 212 | 30 |
| *i* |  |  |  |  |  |  |  | *i* | 385 | 607 | 222 | 29 |  | *i* | 393 | 601 | 208 | 29 |
|  | **245** | **21,030** | **20,785** | **33** | **245** | **21030** |  |  | **12,203** | **20,349** | **8,146** | **20** |  |  |  |  |  |  |
| *d** | 147,324 | 147,612 | 288 | 20 | 147,324 | 147,612 |  | *d** | 147,328 | 147,615 | 287 | 38 |  | *d** | 147,331 | 147,597 | 266 | 18 |
|  | 620,830 | 620,876 | 46 | 10 | 620,830 | 620,876 |  |  | 620,797 | 620,915 | 118 | 17 |  |  | 620,744 | 620,947 | 203 | 7 |
| *e* | 929,279 | 929,439 | 160 | 12 | 4,586,549 | 4,586,389 |  | *e* | 929,330 | 929,450 | 120 | 25 |  | *e* | 929,330 | 929,450 | 120 | 25 |
| *f* | 1,134,090 | 1,134,389 | 299 | 23 | 4,381,738 | 4,381,439 |  | *f* | 1,134,092 | 1,134,389 | 297 | 45 |  | *f* | 1,134,108 | 1,134,370 | 262 | 45 |
| *g* | 1,344,675 | 1,344,936 | 261 | 21 | 4,171,153 | 4,170,892 |  | *g* | 1,344,682 | 1,344,947 | 265 | 40 |  | *g* | 1,344,681 | 1,344,935 | 254 | 40 |
|  | 1,416,134 | 1,416,359 | 225 | 8 | 4,099,694 | 4,099,469 |  |  | 1,416,192 | 1,416,268 | 76 | 22 |  |  | 1,416,190 | 1,416,308 | 118 | 22 |
|  | 1,676,434 | 1,676,469 | 35 | 10 | 3,839,394 | 3,839,359 |  |  | 1,676,523 | 1,676,543 | 20 | 15 |  |  | 1,676,337 | 1,676,542 | 205 | 6 |
| *h* | 3,161,136 | 3,161,279 | 143 | 14 | 2,354,692 | 2,354,549 |  | *h* | 3,161,129 | 3,161,287 | 158 | 27 |  | *h* | 3,161,139 | 3,161,270 | 131 | 27 |
