# Supplementary material for: Regional Control of Chromosome Segregation in Pseudomonas aeruginosa
Source: PLoS Genet. 2016 Nov 7;12(11):e1006428. doi: 10.1371/journal.pgen.1006428 (PMC5098823; doi:10.1371/journal.pgen.1006428)
Supplement: S2 Table — % indicates the percentage of each category among the whole population of cells, whereas italicized numbers between brackets indicate the percentage of each category among cells containing foci. Numbers of cells considered are indicated below. (DOCX) [file pgen.1006428.s008.docx]

**Table S2: Foci number repartition in pPSV38-NGFP-ParB containing strains.** % indicates the percentage of each category among the whole population of cells, whereas italicized numbers between brackets indicate the percentage of each category among cells containing foci. Numbers of cells considered are indicated below.

|  | Δ*parS123* | Δ*parS1234* *parS* +347 | Δ*parS1234* *parS* +552 | Δ*parS1234* *parS* -898 | Δ*parA* |
| --- | --- | --- | --- | --- | --- |
| 0 focus cells (%) | 4 % | 12 % | 10 % | 42 % | 31 % |
| 1 focus cells (%) | 4 % *(4)* | 13 % *(15)* | 16 % *(18)* | 14 % *(24)* | 11 % *(16)* |
| 2 foci cells (%) | 90 % *(94)* | 73 % *(83)* | 72 % *(80)* | 40 % *(69)* | 48 % *(68)* |
| >2 foci cells (%) | 2 % *(2)* | 2 % *(2)* | 2 % *(2)* | 4 % *(7)* | 11 % *(16)* |
|  | *n=1761* | *n=2364* | *n=2349* | *n=3982* | *n=2593* |
