# Supplementary material for: Regional Control of Chromosome Segregation in Pseudomonas aeruginosa
Source: PLoS Genet. 2016 Nov 7;12(11):e1006428. doi: 10.1371/journal.pgen.1006428 (PMC5098823; doi:10.1371/journal.pgen.1006428)
Supplement: S3 Table — (DOCX) [file pgen.1006428.s009.docx]

**Table S1:** Strains used in this study

| Strain number | Strain description | Ref |
| --- | --- | --- |
| IVGB379 | PAO1 ParB-3xFLAG | This study |
| IVGB396 | PAO1 ParB-3xFLAG parST1-PA2258(3,028-R) + pPSV35Ap-TetR-Cfp-yGFP-ParBT1 | This study |
| IVGB397 | PAO1 ParB-3xFLAG parST1-PA0069(82-R) + pPSV35Ap-TetR-Cfp-yGFP-ParBT1 | This study |
| IVGB469 | Δ*parS123* | This study |
| VLB1 | Δ*parS1234* | This study |
| VLB3 | Δ*parS123* + ParB-3xFLAG | This study |
| VLB4 | Δ*parS1234* ParB-3xFLAG | This study |
| IVGB123 | PAO1 parST1-PA2319(2,957-R) tetO-PA0069(82-R) + pPSV35Ap-TetR-CFP-yGFP-ParBT1 | [[1](#_ENREF_1)] |
| VLB13 | Δ*parS123* parST1-PA2319(2,957-R) tetO-PA0069(82-R) + pPSV35Ap-TetR-CFP-yGFP-ParBT1 | This study |
| VLB21 | Δ*parS1234* parST1-PA2319(2,957-R) tetO-PA0069(82-R) + pPSV35Ap-TetR-CFP-yGFP-ParBT1 | This study |
| VLB63 | Δ*parS1234* *parS2* +6.5 | This study |
| VLB62 | Δ*parS1234* *parS9* +6.5 | This study |
| VLB69 | Δ*parS1234* *parS* -898 | This study |
| VLB70 | Δ*parS1234* *parS* -440 | This study |
| IVGB481 | Δ*parS1234* *parS* -330 | This study |
| VLB66 | Δ*parS1234* *parS* +347 | This study |
| IVGB479 | Δ*parS1234* *parS* +449 | This study |
| VLB73 | Δ*parS1234* *parS* +545 | This study |
| IVGB480 | Δ*parS1234* *parS* +552 | This study |
| IVGB317 | Δ*parA* | [[1](#_ENREF_1)] |
|  | Δ*parS1234* PA0290(327-R) + pPSV35Ap-TetR-CFP-yGFP-ParBT1 | This study |
| IVGB478 | Δ*parS1234* *parS* +347 **(VLB66)** parST1-PA0069(82-R) tetO-PA0290(327-R) + pPSV35Ap-TetR-CFP-yGFP-ParBT1 | This study |
| VLB23 | Δ*parS1234* parST1-PA2319(2,957-R) tetO-PA0572(628-R) + pPSV35Ap-TetR-CFP-yGFP-ParBT1 | This study |
| VLB140 | Δ*parS1234* *parS* +552 **(IVGB480)** parST1-PA0069(82-R) tetO-PA0572(628-R) + pPSV35Ap-TetR-CFP-yGFP-ParBT1 | This study |
| IVGB509 | Δ*parS1234* parST1-PA5480(92-L) tetO-PA4822(851-L) + pPSV35Ap-TetR-CFP-yGFP-ParBT1 | This study |
| IVGB510 | Δ*parS1234* *parS* -898 **(VLB69)** parST1-PA5480(92-L) tetO-PA4822(851-L) + pPSV35Ap-TetR-CFP-yGFP-ParBT1 | This study |
| IVGB292 | PAO1parST1-PA0069(82-R) tetO-PA0290(327-R) + pPSV35Ap-TetR-CFP-yGFP-ParBT1 | This study |
| IVGB168 | PAO1parST1-PA0069(82-R) tetO-PA0572(628-R) + pPSV35Ap-TetR-CFP-yGFP-ParBT1 | This study |
| IVGB173 | PAO1parST1-PA5480(92-L) tetO-PA4822(851-L) + pPSV35Ap-TetR-CFP-yGFP-ParBT1 | This study |
| IVGB524 | Δ*parS1234* *parS* -330 Δ*rrnD* | This study |
| IVGB526 | Δ*parS1234* *parS* -898 Δ*rrnD* | This study |
| VLB271 | Δ*parS1234parS attL* 851-L *attR* 82-R | This study |
| VLB272 | Δ*parS1234parS attL* 851-L *attR* 327-R | This study |
| VLB273 | Δ*parS1234parS attL* 851-L *attR* 92-L | This study |
| VLB276 | Δ*parS1234parS attL* 851-L *attR* 82-R INVERTED | This study |
| VLB277 | Δ*parS1234parS attL* 851-L *attR* 327-R INVERTED | This study |
| VLB278 | Δ*parS1234parS attL* 851-L *attR* 92-L INVERTED | This study |
| IVGB556 | Δ*parS1234parS attL* 628-R *attR* 82-R | This study |
| IVGB557 | Δ*parS1234parS attL* 628-R *attR* 327-R | This study |
| IVGB558 | Δ*parS1234parS attL* 628-R *attR* 92-L | This study |
| VLB333 | Δ*parS1234parS attL* 628-R *attR* 82-R INVERTED | This study |
| VLB334 | Δ*parS1234parS attL* 628-R *attR* 327-R INVERTED | This study |
| VLB335 | Δ*parS1234* *attL*-*parS*-PA0572(628-R) *attR*-PA5480(92-L) INVERTED | This study |

1. Vallet-Gely I, Boccard F (2013) Chromosomal organization and segregation in *Pseudomonas aeruginosa*. PLoS Genet 9: e1003492.
