# Supplementary material for: Regional Control of Chromosome Segregation in Pseudomonas aeruginosa
Source: PLoS Genet. 2016 Nov 7;12(11):e1006428. doi: 10.1371/journal.pgen.1006428 (PMC5098823; doi:10.1371/journal.pgen.1006428)
Supplement: S4 Table — (DOCX) [file pgen.1006428.s010.docx]

**S4 Table. Oligonucleotides used in this study**

| **Plasmide** | **Oligo** | **Sequence** |
| --- | --- | --- |
| **pP30D-FRT-ParB-3xFLAG** | DEG5562O1 | AT**AAGCTT**ATGGCCGCTACGAGATCA |
|  | 3xFLAGparBO2 | AT**GGATCC**TCACTTATCATCGTCATCCTTGTAGTCGATGTCATGATCTTTATAATCACCGTCATGGTCTTTGTAGTCaGCGGCCGCACGGATGTGGGCGAGAAC |
| **pEXG2Δ*parS1*** | MparS1O1 | ATAT**TCTAG**AAGGCCGAGGAAGAAGTGC |
|  | MparS1O2 | CAGTTCCTCGATTGGGGAGTTTTTCATGTTGAGCCTCGGTTTCTGCCCGT |
|  | MparS1O3 | ACGGGCAGAAACCGAGGCTCAACATGAAAAACTCCCCAATCGAGGAACTG |
|  | MparS1O4 | AT**AAGCTT**TGGATACCGGCGTATCCG |
| **pEXG2Δ*parS2*** | MparS2O1 | ATATTCTAGATCGGTTTCTGCCCGTCTG |
|  | MparS2O2 | CGGTATCCATGTTTCATGTTGAGCATGGAAAAGTCTCTCAGACCACGACC |
|  | MparS2O3 | GGTCGTGGTCTGAGAGACTTTTCCATGCTCAACATGAAACATGGATACCG |
|  | MparS2O4 | AT**AAGCTT**AGTTGAGGAAGGACAGCT |
| **pEXG2Δ*parS3*** | MparS3O1 | AT**AAGCTT**CCCCGATCCCTGGAAAAC |
|  | MparS3O2 | ATAT**GAATTC**CGAACGCATCCGTCAGGG |
|  | MparS3O3 | ATAT**GAATTC**TCGGCGCGCAGCATAGCT |
|  | MparS3O4 | ATAT**TCTAGA**TGGAAGGTGCGACGCGAT |
| **pEXG2Δ*parS4*** | MparS4O1 | AT**AAGCTT**ATTGATCAGCAAGTTCGT |
|  | MparS4O2 | ATAT**GAATTC**CATCACCGTTCAGGCTTC |
|  | MparS4O3 | ATAT**GAATTC**GCATGAGCCACTGATCAC |
|  | MparS4O4 | ATAT**TCTAGA**CCATGAACGGATTGTCGC |
| **pP30D-FRT-*parSP1*** | ParsP1O1 | AT**AAGCTTGGTACCGCGGCCGC**CTTGTTGTCCACAACCGT |
|  | ParSP1O2 | AT**GGATCCGAATTC**GCGTAAGTTACTCAATTT |
| **pP30D-FRT-*parS2*** | ParS2O1 | ATAT**CTGCAG**CTTGCTTGAAGATTTGGG |
|  | ParS2O2 | ATAT**GAATTC**CTTGATGCTGGAAGAGTC |
| **pP30D-FRT-*parS9*** | ParS9O1 | ATAT**CTGCAG**AGGCGCTTGAGCAGGTCG |
|  | ParS9O2 | ATAT**GAATTC**CAGGTCCAGGTGATGCTG |
| **pP30D-FRT-*parS2-*PA0004 pP30D-FRT-*parS9-*PA0004** | ParS0004O1 | AT**AAGCTT**GAACCCTGAGCAGCTGTG |
|  | ParS0004O2 | AT**GGTACC**CAACGCCGGGCAGTATTG |
| **pP30D-FRT-*parS2-*PA0306** | ParS0306O1 | AT**AAGCTT**CCGATCTCCAGGGTGGCG |
|  | ParS0306O2 | AT**GGTACC**GGGTTGCTACTGCCCCTG |
| **pP30D-FRT-*parS2-*PA0500** | ParS0500O1 | AT**GGTACC**ATCCACAGTTTCCACGCT |
|  | ParS0500O2 | AT**AAGCTT**GACTTCGGACCTGGAAGA |
| **pP30D-FRT-*parS2-*PA4778** | ParS4778O1 | AT**AAGCTT**GCCAGTGCCGACGTCAAG |
|  | ParS4778O2 | AT**GGTACC**TTGGGATGAAAACGGCGG |
| **pP30D-FRT-*parS2-*PA5170** | ParS5170O1 | AT**AAGCTT**GCTGGGCGCGACCATCCG |
|  | ParS5170O2 | AT**GGTACC**GAAATTGCTCGAGATCGG |
| **pEXG2Δ*parS5*** | MparS5O1 | AT**GGTACC**GTGTCGAAGGCCTGGTCG |
|  | MparS5O2 | ATAT**GAATTC**CCGTGTATTCGACCTACCTGCTG |
|  | MparS5O3 | ATAT**GAATTC**CTCCGAAAGGGG |
|  | MparS5O4 | AT**AAGCTT**TCTTCCTTGCTGAACGGC |
| **pEXG2Δ*parS6*** | MparS6O1 | AT**GGTACC**ACCCTGCAACGCCTGGAA |
|  | MparS6O2 | ATAT**GAATTC**CGCCTGGAGTATGCCGGC |
|  | MparS6O3 | ATAT**GAATTC**GGCGAGGCCATCGAGCCG |
|  | MparS6O4 | AT**AAGCTT**CAGTTCGTCGGCGTCGTG |

| **Plasmide** | **Oligo** | **Sequence** |
| --- | --- | --- |
| **pEXG2Δ*parS10*** | MparS10O1 | AT**GGTACC**AATTGTTCCGGCCCCCTC |
|  | MparS10O2 | ATAT**GAATTC**ATCCGGTGCCCTACGAAC |
|  | MparS10O3 | ATAT**GAATTC**GGCAGCCGGTGACCAGCG |
|  | MparS10O4 | AT**AAGCTT**CTCGAGGACTACGACTAT |
| ***parS3* version of the 3 previous plamids** | parS3O1 | ATAT**GAATTC**CTGCGACCGTTACGCCCA |
|  | parS3O2 | ATAT**GAATTC**GAATGCGAGGGCGGCAGC |
| **pEXMΔ*rrnD*** | MRRN4O1 | AT**AAGCTT**CAGCGCCTTCCTTCTTGG |
|  | MRRN4O2 | ATAT**GAATTC**ACGGATGGACGGCTACCT |
|  | MRRN4O3 | ATAT**GAATTC**TCAAATAACCGCCGGAAG |
|  | MRRN4O4 | AT**TCTAGA**ATCTTCATGATGGCGGTG |
| **pP30D-FRT-*parS*-*attL-*PA4822 pP30D-FRT-*attL-parS*-PA0572** | parS3O1bis | AT**AAGCTT**CCTGCGACCGTTACGCCCA |
|  | parS3O2bis | AT**AAGCTT**GAATGCGAGGGCGGCAGC |
| **pCXIPA** | cIup_BglIIbis | ATAT**AGATCT**AGAACACCTTGCCGATCAGC |
|  | Intdo | ACCATGATTACGCCAAGC |
| **pPSV38-NGFP** | GFPNterO1 | atat**GAATTC**GAGGAGGATACATATGGTGAGCAAGGGCGAGGAG |
|  | GFPNterO2 | AT**GGTACC**GCGGCCGCCTTGTACAGCTCGTCCAT |
|  | FNTERParBO1 | atat**GCGGCCGC**aGCAGCCAAGAAACGTGGA |
|  | FNTERParBO2 | AT**AAGCTT**TTTCCGACTACCCGCTAC |
