# Supplementary material for: Regional Control of Chromosome Segregation in Pseudomonas aeruginosa
Source: PLoS Genet. 2016 Nov 7;12(11):e1006428. doi: 10.1371/journal.pgen.1006428 (PMC5098823; doi:10.1371/journal.pgen.1006428)
Supplement: S1 Text — (DOCX) [file pgen.1006428.s011.docx]

**S1 Text**

**Supporting Materials and Methods**

**Plasmids and Strains constructions**

*Escherichia coli* DH5alpha (Invitrogen) was used as the recipient strain for all plasmid constructions, whereas *E. coli* strain β2163 [[1](#_ENREF_1)] was used to mate plasmids into *P. aeruginosa*.

All the integration vectors carry the mobilization region from RP4, the ColE1 origin of replication and the *aacC1* gene (conferring resistance to gentamicin). Plasmids derived from the pP30D-FRT-eGFP [[2](#_ENREF_2)] also contain FRT sites for efficient Flp recombinase-mediated excision. Plasmids derived from the pEXG2 [[3](#_ENREF_3)] also contain the *sacB* gene for allelic exchange.

Plasmids allowing insertion of chromosomal tags and their visualization using fluorescent proteins have been described previously [[2](#_ENREF_2)].

In order to replace the chromosomal gene encoding ParB (PA5562), by a gene encoding a 3xFLAG tagged version of this protein, we engineered plasmid pP30D-FRT-ParB-3xFLAG. Using PCR, we add the sequence encoding the 3xFLAG to the 3’ portion of the *parB* gene, and cloned this fragment into *Hind*III/*Bam*HI cut pP30D-FRT-eGFP. Strains PAO1 ParB-3xFLAG, D*parS123* ParB-3xFLAG and D*parS1234* ParB-3xFLAG were constructed by mating PAO1, Δ*parS123* and Δ*parS1234* with β2163 containing pP30D-FRT-ParB-3xFLAG and selected on *Pseudomonas* isolation agar (PIA) (Difco) containing gentamicin (60 μg/ml) as previously described [[2](#_ENREF_2)]. The plasmid backbone was excised from the PAO1 ParB-3xFLAG through transient synthesis of FLP recombinase from plasmid pFLP2 [[4](#_ENREF_4)]. Resulting strains were then mated with β2163 containing the pP30D-FRT-parST1-PA2258 and pP30D-FRT-parST1-PA0069 plasmids, and transformants were once again selected on PIA containing Gentamicin. In order to visualize the chromosomal tags, plasmid pPSV35Ap-TetR-Cfp-yGfp-ParBT1 was introduced by electroporation, and transformants selected on LB supplemented with Carbenicillin (300 μg/ml).

In order to inactivate *parS* sites, we engineered plasmids pEXG2Δ*parS1*, pEXG2Δ*parS2.* pEXG2Δ*parS3* and pEXG2Δ*parS4* by amplifying flanking regions by the PCR and then splicing the flanking regions together by overlap extension PCR*.* Silent points mutations were introduced in *parS1* and *parS2*: the *parS1* sequence was changed into TTTTTCATGTTGAGCC, and the *parS2* sequence was changed into TGTTTCATGTTGAGCA. The *parS3* and *parS4* sites were replaced by the 6-bp linker sequence 5’-GAATTC-3’. Deletions were confirmed by PCR and sequencing.

Plasmid pP30D-FRT-*parSP1* was engineered by cloning approximately 150 pb containing the *parSP1* site from pGBKD3*parS* [[5](#_ENREF_5)] on a *Hind*IIII/*Bam*HI fragment into pP30D-FRT-eGFP. This fragment was amplified by PCR and *Kpn*I and *Not*I sites were also introduced upstream of *parSP1*, as well as a *Eco*RI site downstream of *parSP1*. Next, we replaced the *Pst*I/*Eco*RI fragment containing the *parSP1* site with approximately 150 pb containing *parS2* and *parS9* from PAO1, resulting into the pP30D-FRT-*parS2* and pP30D-FRT-*parS9* plasmids. Approximately 500 bp of intergenic region were then cloned into these plasmids, giving rise to pP30D-FRT-*parS2-*PA0004*,* pP30D-FRT-*parS9-*PA0004, pP30D-FRT-*parS2-*PA0306, pP30D-FRT-*parS2-*PA0500, pP30D-FRT-*parS2-*PA4778 and pP30D-FRT-*parS2-*PA5170. These plasmids were transformed into β2163 and mated with the Δ*parS1234* mutant*,* and transformants were selected on PIA supplemented with gentamicin, resulting in strains Δ*parS1234* *parS2* +6.5, Δ*parS1234* *parS9* +6.5, Δ*parS1234* *parS* +347, Δ*parS1234* *parS* +545, Δ*parS1234* *parS* -898 and Δ*parS1234* *parS* -330 respectively. Strains Δ*parS1234* *parS* +449, Δ*parS1234* *parS* +552 and Δ*parS1234* *parS* -330 were generated by allelic exchange resulting in the replacement of the putative *parS5*, *parS6* and *parS10* sequences from [[6](#_ENREF_6)] with the *parS3* sequence, using the pEXG2Δ*parS5-parS3*, pEXG2Δ*parS6-parS3* and pEXG2Δ*parS10-parS3* plasmids.

The deletion construct for the *rrnD* was generated by amplifying flanking regions by the PCR and then splicing the flanking regions together by overlap extension PCR, replacing the *rrnD* operon by a 6-bp linker sequence 5’-GAATTC-3’. The resulting PCR products were cloned on *Xba*I/*Hind*III fragments into plasmid pEXG2, yielding plasmid pEXMΔ*rrnD*. This plasmid was then used to create strains Δ*parS1234* *parS* -330 Δ*rrnD* and Δ*parS1234* *parS* -998 Δ*rrnD* by allelic exchange. Deletions were confirmed by the PCR.

The pP30D-FRT-*attL* plasmid was engineered by cloning the 5’part of *lacZ* fused to the *attL* site from plasmid pG6i [[7](#_ENREF_7)] in the *Not*I/*Bam*HI cut pP30D-FRT-eGFP. The *Sal*I fragment containing *5’lacZ* and *attL* was then replaced by the *Sal*I fragment from plasmid pG6i containing the *attR* site fused to the 3’part of *lacZ*, resulting in plasmid pP30D-FRT-*attR*. The 500 pb of intergenic region from pP30D-FRT-tetO*-*PA4822 [[2](#_ENREF_2)] were cloned in the reverse orientation into the pP30D-FRT-*attL* plasmid, resulting in plasmid pP30D-FRT -*attL-*PA4822. Then, 150 bp containing the *parS3* sequence were cloned into the *Hind*III cut pP30D-FRT -*attL-*PA4822, resulting in plasmid pP30D-FRT-*parS*-*attL-*PA4822. The pP30D-FRT- *attL-parS*-PA0572 plasmid was obtained by cloning the intergenic region from pP30D-FRT-tetO*-*PA0572 [[2](#_ENREF_2)] in the reverse orientation into the pP30D-FRT-*attR*, and by cloning 150 bp containing the *parS3* sequence into the resulting pP30D-FRT-*attL*-PA0572 cut by *Kpn*I. The Δ*parS1234* mutant was then mated with β2163 containing these two plasmids, and transformants were once again selected on PIA containing Gentamicin. The plasmid backbone was excised as described above, resulting in strains *parS attL* 851-L and *parS attL* 628-R respectively. Plasmids pP30D-FRT-*attR-*PA0069, pP30D-FRT-*attR-*PA0290 and pP30D-FRT-*attR-*PA5490 (resulting from the cloning of the intergenic regions from pP30D-FRT-tetO*-*PA0069, pP30D-FRT-tetO*-*PA0290 and pP30D-FRT-tetO*-*PA5480 [[2](#_ENREF_2)] into the pP30D-FRT-*attR*) were then transformed into β2163 and mated with *parS attL* 851-L and *parS attL* 628-R. Transformants were selected on Gentamicin, resulting in strains *parS attL* 851-L *attR* 82-R, *parS attL* 851-L *attR* 327-R, *parS attL* 851-L *attR* 92-L, *parS attL* 628-R *attR* 82-R, *parS attL* 628-R *attR* 327-R and *parS attL* 628-R *attR* 92-L respectively.

The pCXIPA was engineered by cloning the *int* and *xis* gene from plasmid pTSA-CXI [[7](#_ENREF_7)] on a PCR amplified *Bgl*II/*Sac*I fragment into the pFLP2 [[4](#_ENREF_4)] cut with *Bam*HI/*Sac*I. This plasmid was then electroporated into the *attL* *attR* containing strains. Blue colonies were the one in which a recombination between *attL* and *attR* upon Int and Xis action lead to the reconstitution of a functional *lacZ* gene (containing a *attB* site in frame), and inversion of the region between *attL* and *attR.* For more details, see [[7](#_ENREF_7)]. The pCXIPA was then counterselected on sucrose. This allowed us to generate strains *parS attL* 851-L *attR* 82-R INVERTED, *parS attL* 851-L *attR* 327-R INVERTED, *parS attL* 851-L *attR* 92-L INVERTED, *parS attL* 628-R *attR* 82-R INVERTED, *parS attL* 628-R *attR* 327-R INVERTED and *parS attL* 628-R *attR* 92-L INVERTED.

The pPSV38-NGFP plasmid was engineered by cloning the PCR amplified eGFP encoding gene (from pP30D-FRT-eGFP, which originate from the pGBM2kan-gfp plasmid [[8](#_ENREF_8)]) in the EcoRI/KpnI cut pPSV38-sspB [[9](#_ENREF_9)]. A Shine-Dalgarno sequence was added upstream of the ATG, and a NotI site was added downstream of the eGFP encoding gene, allowing in frame fusion. The parB gene was then cloned in frame into the NotI/HindIII cut pPSV38-NGFP plasmid, resulting in the pPSV38-NGFP-ParB. Functionality of this GFP-ParB construct was tested by introducing the plasmid into a Δ*parB* mutant and assessing its ability to complement its growth defect.

Oligos are listed in S4 Table and plasmid sequences are available on request.

**Supplemental references**

1. Demarre G, Guerout AM, Matsumoto-Mashimo C, Rowe-Magnus DA, Marliere P, et al. (2005) A new family of mobilizable suicide plasmids based on broad host range R388 plasmid (IncW) and RP4 plasmid (IncPalpha) conjugative machineries and their cognate Escherichia coli host strains. Res Microbiol 156: 245-255.

2. Vallet-Gely I, Boccard F (2013) Chromosomal organization and segregation in Pseudomonas aeruginosa. PLoS Genet 9: e1003492.

3. Rietsch A, Vallet-Gely I, Dove SL, Mekalanos JJ (2005) ExsE, a secreted regulator of type III secretion genes in Pseudomonas aeruginosa. Proc Natl Acad Sci U S A 102: 8006-8011.

4. Hoang TT, Karkhoff-Schweizer RR, Kutchma AJ, Schweizer HP (1998) A broad-host-range Flp-FRT recombination system for site-specific excision of chromosomally-located DNA sequences: application for isolation of unmarked Pseudomonas aeruginosa mutants. Gene 212: 77-86.

5. Espeli O, Mercier R, Boccard F (2008) DNA dynamics vary according to macrodomain topography in the E. coli chromosome. Mol Microbiol 68: 1418-1427.

6. Bartosik AA, Lasocki K, Mierzejewska J, Thomas CM, Jagura-Burdzy G (2004) ParB of Pseudomonas aeruginosa: interactions with its partner ParA and its target parS and specific effects on bacterial growth. J Bacteriol 186: 6983-6998.

7. Valens M, Penaud S, Rossignol M, Cornet F, Boccard F (2004) Macrodomain organization of the Escherichia coli chromosome. EMBO J 23: 4330-4341.

8. Espeli O, Nurse P, Levine C, Lee C, Marians KJ (2003) SetB: an integral membrane protein that affects chromosome segregation in Escherichia coli. Mol Microbiol 50: 495-509.

9. Goldman SR, Sharp JS, Vvedenskaya IO, Livny J, Dove SL, et al. (2011) NanoRNAs prime transcription initiation in vivo. Mol Cell 42: 817-825.
